# Low-field and variable-field NMR relaxation studies of H_2_O and D_2_O molecular dynamics in articular cartilage

**DOI:** 10.1371/journal.pone.0256177

**Published:** 2021-08-25

**Authors:** Andrea Crețu, Carlos Mattea, Siegfried Stapf

**Affiliations:** Fachgebiet Technische Physik II/Polymerphysik, Institute of Physics, Technische Universität Ilmenau, Germany; Rijksuniversiteit Groningen, NETHERLANDS

## Abstract

Osteoarthritis (OA) as the main degenerative disease of articular cartilage in joints is accompanied by structural and compositional changes in the tissue. Degeneration is a consequence of a reduction of the amount of macromolecules, the so-called proteoglycans, and of a corresponding increase in water content, both leading to structural weakening of cartilage. NMR investigations of cartilage generally address only the relaxation properties of water. In this study, two-dimensional (T_1_-T_2_) measurements of bovine articular cartilage samples were carried out for different stages of hydration, complemented by molecular exchange with D_2_O and treatment by trypsin which simulates degeneration by OA. Two signal components were identified in all measurements, characterized by very different T_2_ which suggests liquid-like and solid-like dynamics. These measurements allow the quantification of separate hydrogen components and their assignment to defined physical pools which had been discussed repeatedly in the literature, i.e. bulk-like water and a combination of protein hydrogens and strongly bound water. The first determination of ^2^H relaxation dispersion in comparison to ^1^H dispersion suggests intramolecular interactions as the dominating source for the pronounced magnetic field dependence of the longitudinal relaxation time T_1_.

## 1. Introduction

### 1.1. Cartilage and osteoarthritis

Cartilage is an essential part of the body of vertebrates and it is the tissue that allows them to move freely on land and grow up to hundreds of kilograms and still be mobile. It is the tissue that lines bones in joints and absorbs shocks during locomotion. The shock-absorbing properties of cartilage are dictated by its structure. Cartilage can be viewed as a fiber-reinforced, permeable composite gel within a physiological saline solution [1]. Most of it is composed of extracellular matrix, at around 98% of volume. The major fibrous component of cartilage, type II collagen, provides the tensile strength of cartilage [2]. It has a clearly defined microscopic structure, with a thickness that ranges between 1 and 4 mm. The structure presents distinctive zones due to the orientation of the collagen fibers. These are: the surface zone, where the fibers are located parallel to the surface; the transition zone, where the fibers turn towards an orientation perpendicular to the bone surface [3]; the radial zone, where the collagen fibers achieve a perpendicular orientation towards the surface and the bone; the calcified zone, which consists of partially calcified cartilage and is a transition towards the mineralized subchondral bone. The calcified zone is heterogeneous and composed of collagen, at about a third of the quantity found in the main body of the hyaline cartilage, as well as of hydroxyapatite, the main mineral component of bone tissue [4]. Even though the calcified zone (CZ) plays an important role in the development of osteoarthritis (OA), it is not the main subject of the current investigation, which concentrates on the bulk of hyaline articular cartilage.

The cartilage matrix is synthesized by the chondrocytes, or cartilage cells. These are scarce and have a low activity, which in turn means that cartilage is slow to repair itself after damage. The pores of the collagen matrix are filled with proteoglycan aggregates, which are composed of proteins and aggrecan-hyaluronic acid complexes, generating a high osmotic pressure through electrostatic forces [1]. The proteoglycan assemblies expand against the confining collagen network, producing an osmotically prestressed tissue. This allows cartilage to retain its structure, even under high pressures. If the collagen matrix fails and the proteoglycan molecules are free to move through the tissue or if the proteoglycan molecules become degraded and broken into fragments that are no longer immobilized by the collagen fibers, the tissue loses its properties and its ability to sustain load to the same degree as prior to degradation.

Osteoarthritis (OA) is a progressive disease that affects the hyaline articular cartilage through complex mechanisms, being influenced by multiple factors: age, weight, genetics, activity levels and injuries, as well as the presence of other diseases. It is a degradation of the cartilage in joints which leads to pain of increasing intensity, joint stiffness and even loss of mobility [5]. It impacts people not only on an individual level, but also at the level of society as a whole, from a social and economic standpoint. Various treatments are being tested in an effort to reduce the pain generated by OA, although only after the disease starts to show symptoms [6]. Treatment and prevention could be deployed in the case of early detection of OA through regular screening. Currently there are different screening methods being tested, using invasive techniques, such as analysis of biomarkers in body fluids [7] or X-ray imaging coupled with machine learning [8]. Magnetic Resonance Imaging (MRI) is employed primarily for morphological studies of tissue thickness and joint gap measurements, but the information contained in relaxation contrast is increasingly being addressed [1, 9, 10]. In establishing the relation of relaxation parameters to OA, enzymatic treatment by trypsin is frequently being used as a proxy for tissue degeneration by the disease [11, 12].

### 1.2. Nuclear magnetic resonance approaches

In Magnetic Resonance studies of cartilage, a focus is laid on clinical imaging which is being carried out at magnetic field strengths of typically 1.5 to 3 T, usually providing limited resolution within the cartilage structure of a few mm in thickness; the rather short transverse relaxation times compared to most other tissues in the body constitute a significant problem for *in vivo* MRI. Many fundamental studies of cartilage properties have therefore been performed ex vivo at various magnetic field strengths, in particular with microimaging equipment available at higher fields, allowing much better spatial resolution on the order of 10–20 micrometers [13, 14]. On the other hand, low-field or variable-field studies address particular aspects of relaxation and diffusion properties in some detail [15, 16]. Low-field MRI is a recent addition to the toolbox of investigations of joints.

Apart from spin density imaging, relaxation times and their distributions, as well as measurements of the diffusion tensor in the anisotropic tissue are the most frequently studied parameters. There are several drawbacks to using *ex vivo* samples, such as the fact that tissue excised from a joint does not perform the original function and is no longer under its original constraints, which translates in practice into macroscopic differences, such as changing shape and orientation in space of the excised tissue. These macroscopic changes will affect some of the experimental results. Nevertheless, the microscopic structure and orientation of the fibers themselves are not as affected by the excision or the different mechanical conditions under which the tissue is held, allowing for the in-depth study of the molecular interactions in the complex collagen-proteoglycan matrix. An overview of the current state-of-the-art in NMR and MRI applications to cartilage can be found in [1].

Beyond structural features, the interpretation of relaxation times becomes more and more important in preclinical research for establishing a correlation between measured parameters and severity of the disease, such as osteoarthritis. All biological tissue has complex relaxation behavior; typically, morphological differences within one type of tissue are small, and averaging within a voxel does not lead to strong deviation from monoexponential behavior. However, partial volume effects (e.g. interfaces with bone or vessels, outer limitations of a tumor) may lead to ill-defined relaxation properties. There is a growing awareness of treating such nonexponential signal decays, more frequently in T_2_ where variations and non-exponentialities are often significant at clinical field strengths.

Of all tissues in the body, cartilage possesses the widest variation of T_2_ within a given space of 1–2 mm, owing to the layered structure of clearly separated zones (see below). The variation of T_2_ can amount to a factor of 5 or even more, while at the same time being affected by orientation with respect to the magnetic field direction, potentially broadening the range of values even further. In many MRI protocols, the structure of articular cartilage is spatially not well resolved, and each voxel will typically include spins with a certain range of T_2_ values; a simple exponential fit is insufficient and will deliver inacceptable data quality or even erroneous results. T_1ρ_, which has been suggested in recent years as an alternative diagnostic parameter, has similar properties and shares with T_2_ the dependence on orientation, which counts as a disadvantage in *in vivo* applications where the orientation of a joint with respect to the magnetic field vector often cannot be controlled, and where the curvature of cartilage generates a superposition of orientations, and hence of T_2_ values.

In general, T_2_ shows larger contrast between tissues than T_1_, and is often more suitable as a biomarker for a particular task. However, it has been proven that towards lower magnetic field strengths, the differentiation in T_1_ generally becomes more pronounced as well [17]. In T_2_, quasi-solid components are mostly invisible to conventional hardware, although magnetization exchange may indirectly lead to an influence of the short-T_2_ component to the signal. At the same time, short T_2_ components have been investigated to study the constitution, properties and mobilities of macromolecules [18]. T_1_ and T_2_ distributions show the potential of identifying structural and dynamic detail of tissue, even when they cannot be carried out in vivo. The full dynamics properties will require the use of Fast Field Cycling (FFC) relaxometry, i.e. experiments at variable magnetic field strength (see below).

### 1.3. Low-field relaxation and two-dimensional relaxation experiments

Apart from availability and low cost of equipment working with magnetic field strengths of 1 T and below, a particular advantage is found in the fact that relaxation contrast of T_1_ is generally enhanced towards lower fields. This can be explained qualitatively by the structure of the fundamental equations of the dependence of T_1_ on Larmor frequency ω, or rather the spectral density I(ω), which contains significant contributions at low frequencies in the kHz to MHz regime so that dynamic differences between samples, or regions within one cartilage sample, lead to diverging T_1_ values towards low fields; at the same time, at fields of several Tesla, T_1_ is dominated by fast molecular motions and T_1_ values become much more similar. T_2_, on the other hand, is less dependent on ω, but susceptibility effects, which render the observation of the „true” T_2_ difficult, are proportional to B_0_ and therefore tend to disappear at low fields.

The feasibility of transferring typical NMR studies on cartilage to low magnetic field strength was proven in a number of recent works [15, 16]. Since low-field devices often lack gradient systems and therefore do not provide the option of spatial resolution, the heterogeneous structure of cartilage needs to be taken care of by proper data analysis. While the identification of average relaxation times is of significant value, there appears to be further information in the distribution of relaxation times, which is now routinely analyzed by Inverse Laplace Transform (ILT) or similar algorithms. Rather than determining the complete distribution function, the width of this distribution was found to be suitable to generate a single parameter that can be employed as a biomarker for assessing the state of cartilage tissue [17].

Free and bound water was identified by time-domain studies in articular cartilage [19, 20] and was further subdivided into different pools such as structural, double- and single-bonded water molecules along with free water. Other studies have found similar components and have shown good reproducibility among comparable samples within one experiment; at the same time, there is a significant body of literature which shows quite different results in the occurrence of T_2_ components and their assignment, as described in detail in [1], chapter 18. Data analysis, magnetic field strength, hardware, and source of the samples are possible reasons for this apparent discrepancy.

Carrying out two-dimensional experiments by combining T_1_ and T_2_ encoding is of further assistance to identify and separate individual „pools” of magnetization, and to find possible exchange mechanisms between them. While such approaches certainly date back to the 1980s, the actual 2D representation of such datasets has benefitted from the developments of related 2D versions of the ILT algorithm [21]. T_1_-T_2_ correlation maps [22, 23] have been instrumental in improving the understanding of discrete or distributed components of spin sets in complex systems such as crude oil [24, 25] and food materials [26], or porous media such as rocks [22, 27] and cement paste [28, 29] and even biological applications [30]. However, T_1_-T_2_ studies of biological matter are scarce, and the authors are aware of only one recent work where cartilage has been investigated as part of a larger study [31]. This type of correlation study is thus a key element of the presented work, with the aim of identifying the respective role and behaviour of individual relaxation components as a function of hydration, D_2_O replacement and trypsin treatment. In this respect, we follow earlier attempts to identify the different components in multiexponential ^1^H relaxation in cartilage [19, 20, 32], and suggest the 2D approach for providing more detailed insight into this topic. Note that in this paper, we will use the expression „free” and „bulk-like” water with the following meaning, in accordance with literature: „bulk-like” refers to water with relaxation and diffusion properties identical to those of bulk water in the absence of interfaces; a water molecule can have a bulk-like environment at a given moment time but experience surface interactions or exchange with other molecules so that the resulting measured relaxation time will be different from bulk properties. The notion of „free” water refers to molecules freely mobile, but possibly experiencing the presence of salts or contaminants, in contrast to those molecules being in contact to an interface; again, both pools can exchange depending on the timescale.

### 1.4. Single-sided NMR

**Fig 1:**
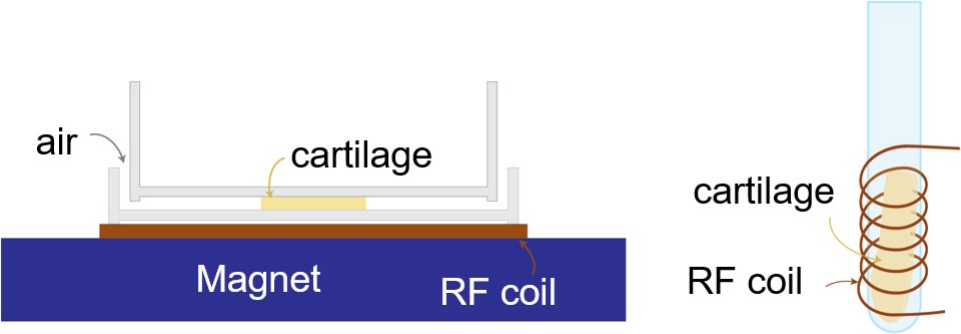
Sample shape and placement for different experiments: a) flat, square sample on top of the magnet, between plastic Petri dishes, b) cylindrical sample in a 5 mm NMR tube in the sensitive area of the NMR coil.

Single-sided NMR scanners allow direct contact of the resonator with a flat surface, such as a wall, a patient’s skin or any object in the vicinity of the receiver coil [33]. A widespread concept of single-sided scanners is the MObile Universal Surface Explorer, or NMR-MOUSE [34] with a flat receiver coil placed above a set of permanent magnets, where the coil’s normal axis is perpendicular to the magnetic field direction. The signal is obtained from within a slice that is given by the size and resonance properties of the coil matching the field conditions within a sweet spot, the thickness of which is determined by the static magnetic field gradient that is aligned along the coil normal axis. For a flat slice, the NMR-MOUSE is most suitable for objects with a depth structure that is homogeneous within the sample itself or within the region of the sweet spot, i.e. the sensitive region of the coil which is typically between 20 and 100 mm^2^, whichever is smaller (see Fig 1). The layer structure of mammalian cartilage was found to meet the conditions required for obtaining a depth scan with the NMR-MOUSE: with a commercial product of a field strength of 0.27 T and a maximum magnetic field gradient of 11.5 T/m, a best resolution of 20 μm was achieved [35]. Curvature and other imperfections of the cartilage necessarily lead to partial averaging of the layer structure, but were found to be acceptable for the main joints of larger mammalians such as bovine or human hip and knee joints. The variation of T_1_, T_2_ and the diffusion coefficient D as a function of distance from the surface were studied [15, 16]. In this work, the single-sided scanner is primarily used for monitoring the drying process of cartilage samples exposed to air and its shrinkage, in order to compare results with those obtained in the absence of spatial resolution in partially de- and rehydrated samples.

### 1.5. Variable-field relaxometry

Fast field-cycling (FFC) relaxometry–as opposed to mechanical sample shuttling between different magnetic fields–uses electronic field switches of a resistive magnet in order to allow the determination of relaxation times at different Larmor frequencies [36]. Since relaxation is a consequence of a superposition of molecular reorientations in a potentially complex environment, covering a broad fraction of the full spectral density function I(ω) allows the comparison with appropriate models for relaxation processes. Out of the multitude of applications, studies of the dynamics of polymers [37, 38] and of liquids in porous media [39, 40] are of particular relevance to the properties of relaxation in cartilage. While cartilage resembles a porous medium, with water molecules moving relatively freely between obstacles of collagen fibers and proteoglycan macromolecules, these water molecules are also affected by their respective mobility due to strong molecular interactions, so that their motion is also modulated by the slow dynamics of the macromolecules themselves. The situation is comparable to hydration water dynamics in protein solutions [41] or hydrogels [42, 43]. However, despite several general approaches to the problem [41, 44–47], a satisfactory description of water relaxation dispersion in cartilage, or any other biological tissue [48], is not yet available. In this work, a focus is put on, for the first time, determining the relaxation dispersions for both water protons and D_2_O deuterons with the aim of simplifying the choice of possible relaxation contributions. Since deuterons relax via quadrupolar interactions, only intramolecular contributions to their reorientations are of relevance, allowing for a possible isolation of intra- and intermolecular contributions in ^1^H relaxation which is predominantly dipolar. For the particular system of nanoporous inorganic glasses, the dominance of intramolecular (i.e. rotational) reorientations was already proven [49].

The typically accessible field range of FFC devices of about 0.2 mT to 1 T (corresponding to approximately 10 kHz to 40 MHz of ^1^H Larmor frequency, or from 1 kHz to 5 MHz ^2^H Larmor frequency, respectively) extends towards a regime where a direct detection of the thermally polarized magnetization would be inconvenient or require excessively large sample volumes. However, this regime is of special interest for cartilage studies since the slow molecular reorientation processes become particularly well observable at these Larmor frequencies, and the difference between “free” and “adsorbed” water dynamics becomes more obvious. In fact it has been suggested that the width of the T_1_ distribution in cartilage assumes a maximum near 20 mT [17, 50]. On the other hand, the frequency dependence of T_2_ is less pronounced, so that all essential information about T_2_, which is dominated by the slowest molecular dynamics processes due to its formula containing a zero-frequency term, can be acquired at an arbitrary magnetic field strength.

Apart from giving access to the full spectral density function of reorientations over 3–4 orders of magnitude, FFC devices also cover the particularly interesting region where a cross-over with the three energy levels of the ubiquitous ^14^N nuclei takes place. This quadrupolar nucleus, with its short intrinsic relaxation time, acts like a relaxation sink for the hydrogen nuclei in its immediate vicinity, provided their interaction is not averaged out by sufficiently fast and isotropic motion. ^14^N nuclei located within the amino acids of proteoglycans, but also of collagen, affect the nuclei of water molecules coming into close contact with them, and fast water exchange leads to a noticeable decrease of the averaged water proton T_1_ relaxation times in three different frequency ranges centered at about 0.6, 2.3 and 2.9 MHz for ^1^H (“quadrupole dips” of the relaxation times). The depth and width of these dips have been identified early as markers for the concentration of ^14^N nuclei and of their mobility [51, 52]. For instance, it was shown that the high mobility of the fibrin monomer does not give rise to quadrupole dips in the surrounding water phase but that these dips occur after polymerization to the more immobile fibrin polymer [53]. Osteoarthritis, as well as treatment with trypsin, destroys part of the proteoglycan (PG) network and reduces the macromolecular concentration, whereas short strands of freely diffusing proteoglycans do not contribute to the quadrupole dips due to their fast isotropic motion. The intensity of the dips decreases with severity of OA, i.e. with the decrease of PG and with the simultaneous increase of water content, both of which are characteristic for the disease [54]. While a negative correlation of quadrupolar dip intensity and OA severity has been found [15, 16, 54], model experiments with trypsin treatment suggest that the influence of water content may be at least as important as the reduction of the ^14^N nuclei content that takes place at the same time [15, 16, 55].

### 1.6. D_2_O measurements

In order to facilitate assignment of the different contributions of ^1^H relaxation times, partial or complete replacement of H_2_O by D_2_O has been attempted sporadically in the literature with *ex vivo* experiments on cartilage [20, 56]. Repeated dilution eventually leads to an almost complete replacement of water molecules by D_2_O (or rather HDO, since fast hydrogen exchange ensures that the remaining small number of protons will distribute into different molecules). What eventually remains are ^1^H nuclei that are unable to exchange, i.e. water molecules rigidly bound or hidden in isolated pockets, or non-exchanging protons within the matrix (PG, collagen, cells). This approach has been used to identify and quantify the different ^1^H pools, whereas partial dilution in D_2_O allows for a distinction of the different relaxation mechanisms. As mentioned above, the relaxation properties of the ^2^H nuclei can likewise be determined, although many low-field and single-sided NMR scanners are not designed for this task. So far, relaxation studies on ^2^H have not yet been carried out, but these would be desirable since they can help to discriminate between the dominating relaxation mechanisms for the molecules involved: ^2^H nuclei relax predominantly by quadrupolar relaxation and therefore probe only intramolecular interactions such as molecular rotation; ^1^H experience contributions from intra- and intermolecular processes.

In this study we present the correlation between T_1_ and T_2_ relaxation times distributions over a particularly wide range of relaxation times, including the contribution from solid-like fractions that are frequently discarded from analyses, for natural bovine articular cartilage samples during drying, rehydration and deuteration, with or without prior trypsin treatment, and support these data by the first ^2^H relaxometry measurements that allow the assignment of the dominating relaxation processes. Understanding the qualitative and quantitative changes, as well as the effects of enzymes such as trypsin on the transverse relaxation values and the dispersion of longitudinal relaxation rate values in bovine articular cartilage provide a firm base for future measurements on human samples. The longitudinal and transverse relaxation values in bovine articular joint cartilage have been extracted at different dehydration stages and after rehydration with different solutions (with varying concentrations of H_2_O and D_2_O) to reveal the dynamics of water molecules within the cartilage matrix, as well as the exchange processes that happen between the matrix and the bulk-like water molecules. Understanding how the state of hydration of the cartilage samples affects the resulting dispersions of relaxation rates will lead to a better data interpretation of results obtained from human articular cartilage samples, either *ex vivo* in laboratory experiments, or *in vivo*, using a whole-body Fast Field-Cycling scanner [57, 58].

## 2. Samples and experiments

### 2.1. Sample preparation

Bovine cartilage was extracted from synovial joints of cows of different age, 24 hours after they were butchered. All samples were obtained from a local abattoir following scheduled slaughtering, and no animal was sacrificed for this study. Samples of cartilage were prepared for different experiments, using three instruments. Two types of experiments were performed: single-sided profile measurements, where the samples are left in their natural shape and environment, and conventional NMR measurements, where the samples were sliced and placed into NMR tubes with a 5 mm diameter, which were subsequently positioned inside the spectrometer resonators. Two types of NMR setup were used for these experiments, with different sample preparation protocols owing to the geometry of the sensitive area of each instrument.

#### 2.1.1. Relaxation measurements at B = 1.0 T—drying experiment

Slices of cartilage (calf, 6–9 months old) were cut from larger pieces with a uniform thickness of about 2.5 mm. They were weighed and placed into 5 mm NMR tubes. Measurements were performed, then the cartilage was extracted, weighed again, allowed to dry in ambient conditions (20°C and 40% ambient humidity, constant ventilation in a fume hood) for between 10 and 60 minutes until significant weight loss was expected, then weighed again and placed inside a clean tube. The procedure was repeated four times, until the sample weight did not change noticeably, this sample was tentatively called “dry”. The procedure resulted in five experiments, labeled D1-D5, where D1 represents the original cartilage and D2 to D5 denote the subsequent drying stages. After drying out completely, the sample was placed in a phosphate buffer solution to rehydrate partially, patted dry on a clean tissue paper, weighed, and measured again, then once more allowed to hydrate to complete saturation. This sample was labeled Re1 (partly rehydrated) and Re2 (completely rehydrated). Three more samples were treated the same way, with similar results.

#### 2.1.2. Relaxation measurements at B = 1.0 T—D_2_O rehydration experiment

For this experiment, slices of cartilage (cow, 30 months old) were cut from the same tissue and weighed. All slices were taken from neighboring regions in the joint, so that their structural characteristics and relaxation times are similar. The slices were cut to similar sizes, weighed, and treated according to the following table (Table 1). Some were measured directly after cutting, some were placed in a PBS solution containing 0.5 mg/ml trypsin, and the remaining samples were allowed to dry in ambient conditions (20°C and 40% humidity). After 24 h, the samples kept in the trypsin solution were washed in fresh PBS solution to remove any mobile phase and any remaining enzymes from the structure. They were transferred to another fresh reservoir of phosphate buffer solution (PBS) for 3 hours and placed on an agitator. After weighing, they were likewise left to dry. For testing, each sample was placed in a 5 mm NMR tube and sealed with a lid.

**Table 1:** Sample labels, procedures and weights for each sample (reh = rehydrated with PBS solution).

Nr.	Sample code	Original mass (g)	Procedure 1 (p1)	Mass after p1	Procedure 2 (p2)	Mass after p2	Procedure 3 (p3)	Mass after p3
1	1Cw	0.1235	none	-	-	N/A	-	-
2	2Cd0	0.0777	drying	0.0175	reh 0% D_2_O	0.0865	-	-
3	3Cd0	0.1182	drying	0.0257	reh 0% D_2_O	0.1285	-	-
4	4Cd0	0.0551	drying	0.0126	reh 0% D_2_O	0.0616	-	-
5	5Cd08	0.0845	drying	0.0161	reh 80% D_2_O	0.0988	-	-
6	6Cd08	0.0651	drying	0.0135	reh 80% D_2_O	0.0794	-	-
7	7Cd08	0.0924	drying	0.0192	reh 80% D_2_O	0.098	-	-
8	8Cd09	0.0757	drying	0.0173	reh 90% D_2_O	0.0912	-	-
9	9Cd09	0.0718	drying	0.0129	reh 90% D_2_O	0.0864	-	-
10	10Cd09	0.0748	drying	0.0144	reh 90% D_2_O	0.0847	-	-
11	11Cd1	0.051	drying	0.0065	reh 100% D_2_O	0.0658	-	-
12	12Cd1	0.0632	drying	0.0128	reh 100% D_2_O	0.0732	-	-
13	13Cd1	0.0649	drying	0.0128	reh 100% D_2_O	0.069	-	-
14	1Tw	N/A	trypsin	0.0524	N/A	-	-	-
15	2Tw	N/A	trypsin	0.066	N/A	-	-	-
16	3Td0	N/A	trypsin	0.0559	drying	0.0092	reh 0% D_2_O	0.0449
17	4Td0	N/A	trypsin	0.0465	drying	0.0085	reh 0% D_2_O	0.045
18	5Td0	N/A	trypsin	0.0712	drying	0.0142	reh 0% D_2_O	0.0684
19	6Td1	N/A	trypsin	0.0556	drying	0.008	reh 100% D_2_O	0.0423
20	7Td1	N/A	trypsin	0.0551	drying	0.0109	reh 100% D_2_O	0.0373
21	8Td1	N/A	trypsin	0.0595	drying	0.0082	reh 100% D_2_O	0.0334

#### 2.1.3. Drying measurement at 0.27 T with the NMR MOUSE

A 1 cm^2^ square cartilage sample, 1.2 mm in thickness, called sample M1, was excised from a flat part of the bovine joint (calf, 6–9 months old, same specimen as in 2.1.1). It was placed between the lid and the bottom of a thin plastic Petri dish (both with the opening facing up). The thickness of the bottom of the Petri dish is 1.8 mm, as can be observed directly from the MOUSE measurements. The sample was covered on both top and bottom, which prevented the water from evaporating too quickly, leaving only the 1.2 mm side of the cartilage in contact with air at ambient temperature of 20°C and 40% humidity, as is sketched schematically in Fig 1A.

#### 2.1.4. Samples for Fast Field cycling (FFC) measurements

One slice of cartilage (calf, 6–9 months old, same specimen as in 2.1.1) was cut from tissue and weighed, then placed inside a 5 mm glass NMR tube that fits into the coil of the NMR instrument (see Fig 1B). A dispersion profile was measured directly after cutting, at 5°C, labeled DF1. After the experiment, the sample was removed from the tube and left to dry in ambient conditions (20°C and 40% humidity) for 20 minutes, then weighed again and placed into a clean tube. Another dispersion profile was measured at the same temperature. This measurement was labeled DF2. The last dispersion profile was performed after the sample was dried until there was no further change in mass. The last measurement was labeled DF3.

Two more slices of cartilage (cow, 30 months old, same specimen as in 2.1.2) were cut from fresh tissue and weighed. One was left to dry in ambient conditions (20°C and 40% humidity) until no further changes were observed in the weight of the sample, then it was placed in D_2_O for rehydration. The other slice was placed in D_2_O (99.9% purity, Sigma Aldrich) for 24 hours for complete exchange, then placed in fresh D_2_O for a further 24-hour exchange period to remove remaining H_2_O molecules. Both samples were weighed after the replacement with D_2_O and then were placed into NMR tubes. The experiments were performed using the ^2^H probe of the instrument.

### 2.2. Experimental details

#### 2.2.1. Relaxation measurements at B = 1.0 T

Two-dimensional measurements of the longitudinal and transverse relaxation times, T_1_-T_2_, of ^1^H nuclei were carried out on a Spinsolve benchtop NMR spectrometer (Magritek, Wellington, New Zealand) with a magnetic field strength of 1.0 T operating at a proton resonance frequency of 43 MHz. Results of these experiments were verified by separate measurements of *either* T_1_ *or* T_2_ distributions. The pulse sequence consists of an FID (free induction decay), followed by a CPMG (Carr-Purcell-Meiboom-Gill) echo train [23] of 13 μs length of the 180° pulses, for measuring transverse relaxation, which was preceded by different recovery intervals of an inversion recovery experiment, for measuring longitudinal relaxation. The FID acquisition allows determination of the transverse relaxation components well below the echo time of the CMPG sequence and thus extends the range of accessible T_2_ values by more than one order of magnitude when compared to conventional echo experiments. The resulting two-dimensional data is processed via an Inverse Laplace Transform (ILT) using a kernel containing a Gaussian and an exponential component [59–61], which produces a T_1_-T_2_ correlation map [22]. In the initial inversion recovery interval, a minimum of 32 recovery times were spaced logarithmically from 1 ms to the maximum value of 5000 ms, which exceeds 5 times the longest T_1_ encountered for the samples rehydrated in H_2_O; for the samples rehydrated in D_2_O, these values were doubled. The waiting time between experiments was equal to the longest recovery interval. The echo time and the number of echoes for the measurements were selected for each hydration stage according to the amount of free water in the sample. To acquire the full transverse relaxation curves, the experiments started at 250 μs echo time and 2000 echoes and decreased to echo times of 100 μs and 100 echoes when the sample was completely dry, since subsequent echoes rendered only noise. The number of accumulations was set between 4 and 8 depending on the actual signal-to-noise ratio. The absence of background signal was verified by performing an experiment on an empty tube with identical parameters.

#### 2.2.2. Drying measurement at 0.27 T with the NMR MOUSE

T_1_ profile measurements during drying were carried out on an NMR-MOUSE single-sided scanner (Magritek, Aachen, Germany) operating at a ^1^H resonance frequency of 11.7 MHz proton resonance frequency and providing a constant magnetic field gradient of 11.5 T/m within the sensitive volume. One-dimensional profiles were measured by moving the sensitive slice vertically through the sample in steps of 100 μm, one complete profile taking around 40 minutes. A profile measurement was performed once every hour, for a total of 20 hours, and consisted of a saturation recovery period followed by acquisition by a CPMG train of 1024 echoes with a spacing of 67.2 μs, where the 180° pulse length was 3.5 μs. Each saturation recovery experiment consisted of 20 time delays points, acquired with 16 scans, to a maximum of 2000 ms, which exceeded 5 times the maximum T_1_ measured, guaranteeing full recovery of the magnetization between acquisitions. An exponential decay function was used to fit each saturation recovery curve.

#### 2.2.3. Fast Field Cycling (FFC) measurements

Relaxation dispersion measurements were performed employing a Stelar Spinmaster (Stelar s.r.l., Mede, Italy) FFC relaxometer, using a protocol that consists of three magnetization evolution intervals: polarization, relaxation, and detection [36]. Polarization took place at a field strength of about 0.3 T (12 MHz ^1^H resonance frequency) whereas detection occurred at 11 MHz Larmor frequency. The polarization time was selected dynamically, based on the relaxation time at each relaxation field, with a duration of 5 times the longitudinal relaxation time (T_1_) at the respective field. In between, the field was switched to relaxation field values corresponding to ^1^H Larmor frequencies between 10 kHz and 21 MHz, with 8 accumulations for each time value. The repetition time was set to exceed 5 times the estimated T_1_, to allow the system to relax to equilibrium before starting the subsequent measurement. For the entire dispersion curve, 25 frequency points were selected in a logarithmic spacing, with a supplementary set of 35 points in the frequency range 1.41–3.5 MHz in order to determine the quadrupolar peaks [16, 52, 62] at high resolution. Relaxation curves were fitted by monoexponential decays, rendering an average value of T_1_ for each Larmor frequency. One profile with the same parameters was measured for each of the three states of the cartilage sample.

Measurements of the ^2^H relaxation dispersion were carried out using a separate probe tuned for ^2^H resonance in a similar range of magnetic field, rendering ^2^H Larmor frequencies between about 1 kHz and 4 MHz. These experiments covered 20–30 logarithmically spaced relaxation fields and employed 16 accumulations for each time value. In all experiments, only one relaxation component was observed due to the dead-time of the relaxometer that rendered the short T_2_ signal component invisible.

## 3. Results

### 3.1. Cartilage drying

#### 3.1.1. Two-dimensional T_1_-T_2_ maps at B = 1.0 T

**Fig 2:**
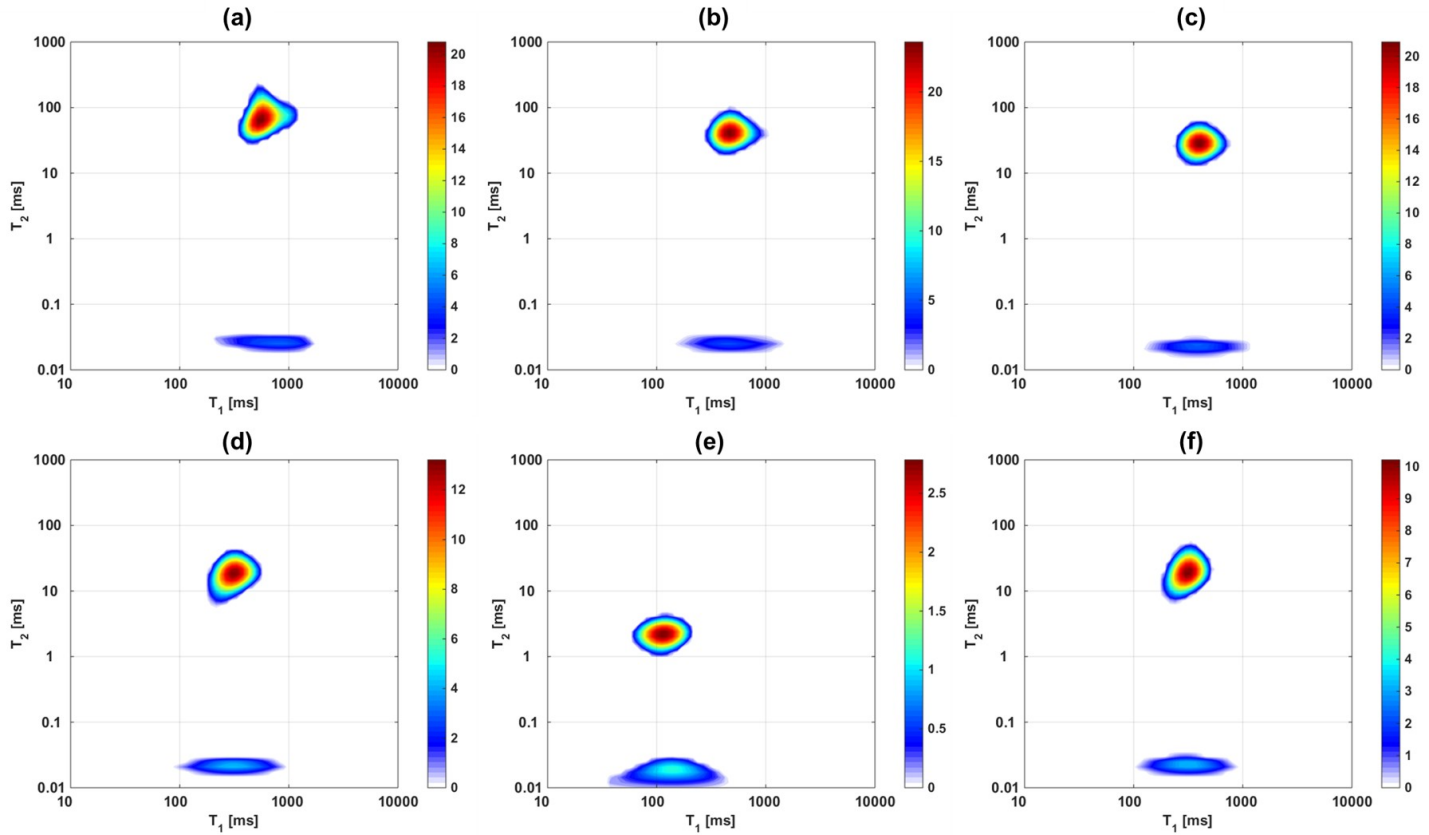
^1^H T_1_-T_2_ maps of one cartilage sample as it undergoes drying and then rehydration: a) D1, b) D2, c) D3, d) D4, e) D5, the mass remains constant after this stage, f) Re1. The secondary peak (at shorter T_2_ times) appears and increases in relative intensity as the main peak decreases in intensity and shifts towards lower T_1_ and T_2_ values.

**Fig 3:**
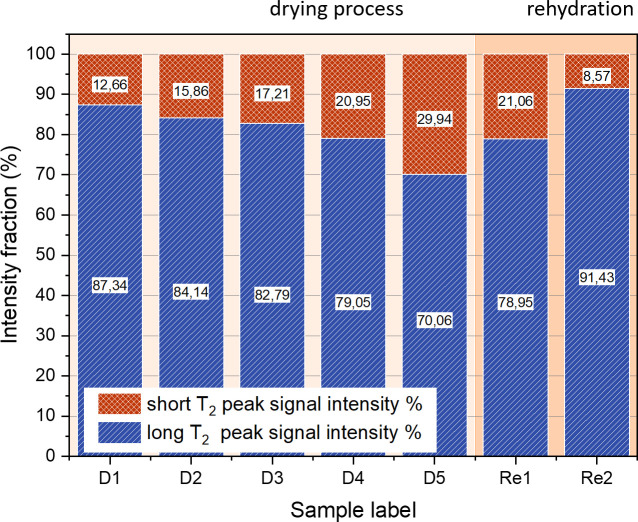
Relationship between the intensities of the two peaks identified in the ^1^H T_1_-T_2_ maps, as the sample is dried and rehydrated.

**Fig 4:**
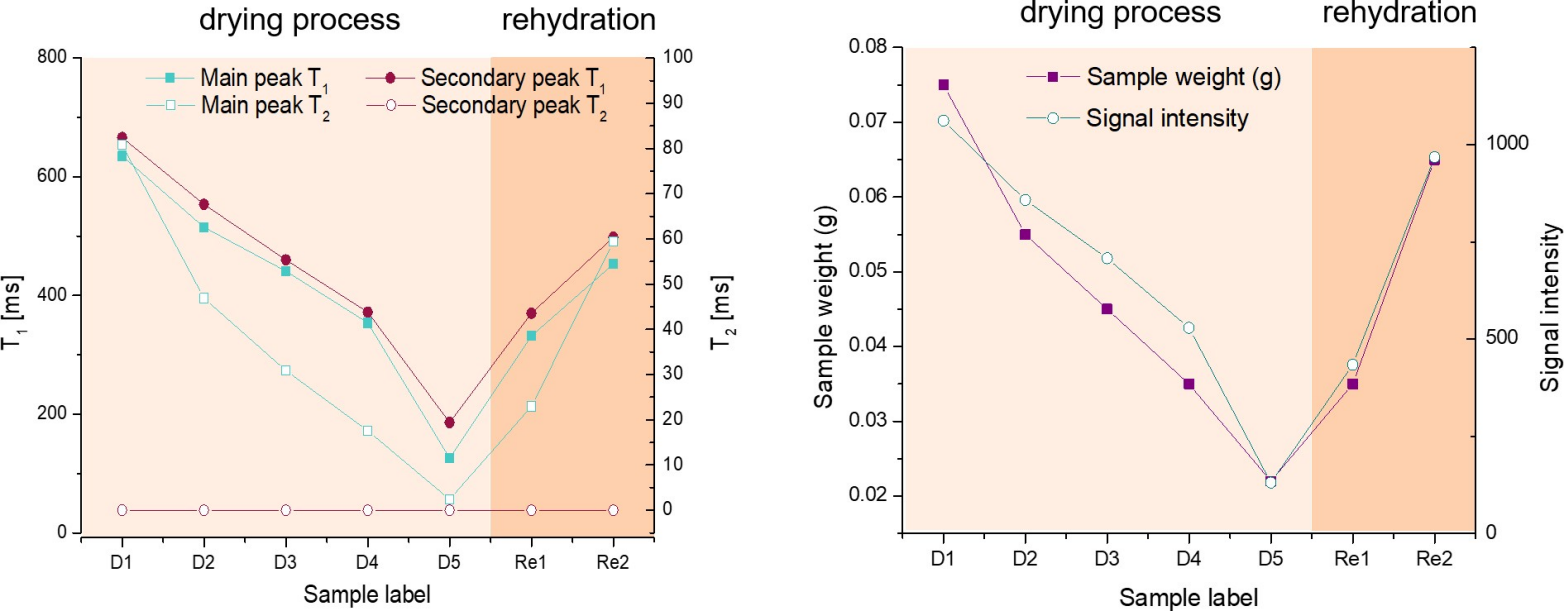
(left) Evolution of ^1^H T_1_ and T_2_ values for both peaks over the whole range of the experiment. The left Y axis represents the T_1_ values, the right Y axis represents the T_2_ values. The values of the secondary peak T_2_ are almost constant at about 20 μs. (right) Evolution of sample weight and signal intensity (data from Table 2).

In Fig 2 the influence of free water removal from the cartilage matrix on the T_1_-T_2_ correlation maps is shown. T_1_ and T_2_ values of the main peak decrease as the cartilage dries, while a second component becomes more prominent at much shorter T_2_ values. This component is frequently absent from experiments carried out with MRI hardware since it only occurs in the FID part of the pulse sequence used in this study, and would decay within the dead time of many standard receiver coils, especially imaging equipment. The T_1_ distribution values, on the other hand, remain within the range between about 100 and 1000 ms and retain a similar width which is comparable for both peaks, possibly somewhat broader for the short T_2_ peak. Table 2 summarizes the sample weights and the respective peak positions and relative fraction of the two peaks, the first obtained from a weighted integral of each peak in the 2D plot, the second derived from the corresponding fraction of the time-domain signal before application of the ILT. The values and the ratio of contributions are additionally visualized in Figs 3 and 4. Values and weight fraction of the short T_2_ component must be considered approximate since a certain fraction of the signal is still expected to be lost during the probe’s dead time of about 15 μs, leading to an underestimation of the weight fraction of this peak and the disregard of possibly existing even shorter T_2_ contributions. It can be estimated that for a deadtime of 15 μs an effective transverse relaxation time of 20 or 30 μs, respectively, will lead to a signal decay of 43% or 22%, respectively, if one assumes a Gaussian signal shape; the maximum error in quantifying the short component between different samples will therefore be in the range 10–20% since T_2_ is certainly not shorter than 20 μs.

**Table 2:** T_1_ and T_2_ values of ^1^H nuclei for the two peaks identified via 2D ILT in all cartilage samples, from completely hydrated, to completely dry, then rehydrated (Re1, Re2).

Sample label	Sample weight (g)	Signal intensity	Main peak			Secondary peak		
			T_1_ (ms)	T_2_ (ms)	intensity	T_1_ (ms)	T_2_ (ms)	intensity
**D1**	0.075	1062	635	80.8	928	666	0.03	134
**D2**	0.055	858	515	46.9	722	554	0.02	136
**D3**	0.045	708	441	30.9	586	460	0.02	122
**D4**	0.035	529	354	17.6	418	372	0.02	111
**D5**	0.022	131	126	2.4	91.8	186	0.02	39.2
**Re1**	0.035	434	332	23	343	370	0.02	91
**Re2**	0.065	968	453	59	885	498	0.02	83

It appears straightforward to assign the component with long T_2_ to the freely moving water that dominates in cartilage. Intermediate components of several ms, as have been reported in some studies ([63, 64] and [1] Chapter18), however, are absent in these measurements. The short T_2_ component, on the other hand, can be assigned to strongly bound water, or protons in macromolecules. A comparison of relative intensities allows a tentative identification:

From the values in Table 2, the free water, or rather “removable water”, can be assigned to the weight difference between the dry weight and actual weight of the sample. The “dry” state, on the other hand, incorporates a certain amount of strongly bound water which would be partially removed only at elevated temperature or under vacuum. Several studies have attempted to quantify the different components of water, and in all situations, a certain amount is considered irreplaceable, being rather rigidly bound to the macromolecules or trapped in molecular-sized pockets [20]. Clearly, the samples in this study have not been dried to this residual water content, so that a fraction of removable water molecules may contribute to the short T_2_ component. A strong positive correlation of the long component of both relaxation times, T_1_ and T_2_, with the sample weight is observed. Note that the T_1_ of the fully rehydrated sample is somewhat lower than the value expected at that total sample weight. This can tentatively be explained by the assumption that the cycle of de-/rehydration does not recover the original situation of water distribution (see Discussion).

#### 3.1.2. MOUSE measurements during drying at B = 0.27 T

**Fig 5:**
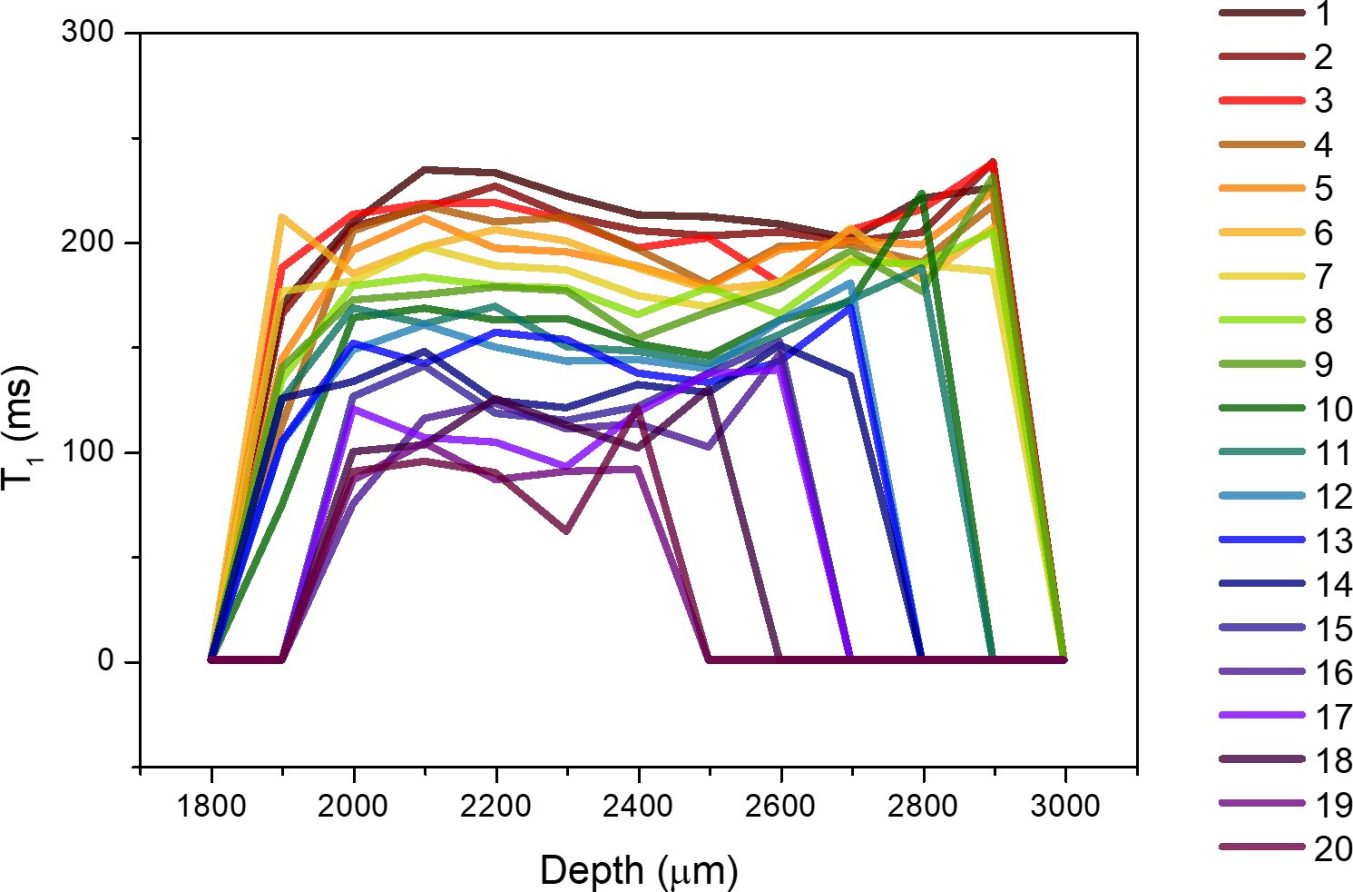
^1^H T_1_ profiles through the drying cartilage as 20 hours pass and the cartilage dries completely. Notice both the decrease in T_1_ values (to less than half) and the shrinkage of the sample in height, as the water is evaporated. The scale is set to zero at the MOUSE sensor’s upper surface, the left-hand limit of the signal corresponds to the bottom of the sample which is on top of the 1.8 mm thick Petri dish.

In order to visualize structural changes of the cartilage sample and identify heterogeneities in drying, sample M1 has been monitored during drying on top of the MOUSE instrument. Saturation recovery experiments at different depths inside the sample reveal the variation of T_1_ values throughout the whole thickness of the tissue [15, 16, 35, 65]. The values of T_1_ across the whole thickness of the sample are shown in Fig 5 for 20 hours of drying. During the first nine hours of drying, T_1_ decreases slowly across the whole thickness of the sample, from values around 230 ms to around 180 ms, as the water evaporates from the whole tissue. No structural changes are observed during these hours. After nine hours, the edges of the profile retreat as the piece of cartilage shrinks. The height of the sample contracts to 0.6 mm, half of its initial height, while T_1_ continues to decrease to values less than half of the original. After 20 hours, the sample is completely dry (i.e. it does not possess any more “free” water) and the T_1_ throughout is about 90 ms. No further changes in the values of T_1_ have been observed for times exceeding 20 hours, which is in agreement with the evaporation of all free water. The state of this sample is thus comparable with that of sample D5 in the previous experiment. The variation of T_1_ during drying is significantly smaller than for sample D at 1.0 T, which can be assigned to the different animal age. Note that T_1_ values are not directly comparable due to their strong field dependence; average T_1_ were found to be about two times shorter at 0.27 T compared to 1.0 T [55].

#### 3.1.3. Field-cycling relaxometry measurements

**Fig 6:**
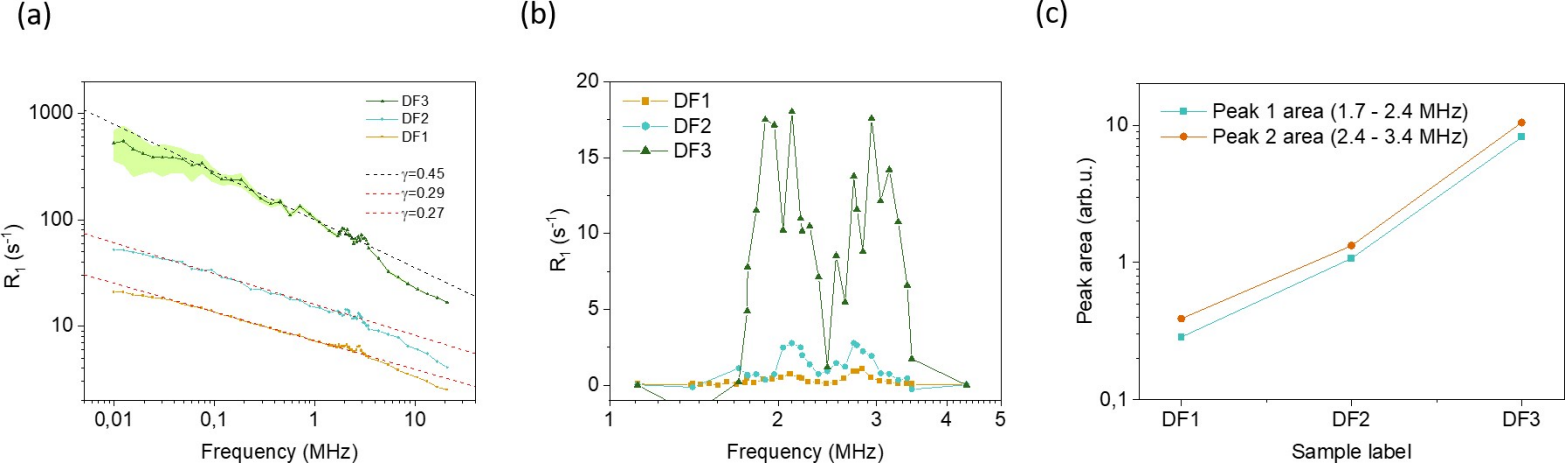
a) ^1^H spin-lattice relaxation profiles (dispersion curves) of the cartilage sample measured at different hydration values, DF1-3, showing the variation in R_1_ values as a function of Larmor frequency as well as the standard deviations that are becoming visible only in the dry sample. Best fits to the lower-frequency power-law exponents are indicated by dashed lines. b) The quadrupolar peaks isolated from the dispersion profiles, with the baseline subtracted. c) The area of the quadrupolar peaks showing a larger increase as the sample becomes less hydrated and the influence of the solid matrix increases.

Field-cycling relaxation measurements were performed to reveal the nature of the interactions of the water molecules with the solid matrix via the frequency dependence of their relaxation times. The dead time of the spectrometer does not allow acquisition of the short component as discussed in 3.1.1. Therefore, the signal stems from the long T_2_ component found at 43 MHz but represents an average across the whole sample tissue. It has been shown before that due to the superposition of different relaxation times from the three layers within cartilage, but also from non-exponential relaxation on a microscopic level in the tissue, a monoexponential fit renders an average value, and the width of the T_1_ distribution, which is still rather small at 43 MHz Larmor frequency, grows with decreasing frequency [17, 50]. Discussing the general shape of the dispersion must therefore remain semi-quantitative, but three observations can nevertheless be made upon sample drying which have not been reported before (see Fig 6A): (i) the relaxation rate increases at all field strengths; (ii) the frequency dependence, i.e. the slope in the plot of Fig 6A, increases for the sample with least water content; (iii) the area of the quadrupolar dips increases. Observation (i) is fully equivalent to the results obtained at 1.0 T. Assuming that slow (longer than 10^−8^ s, given by the inverse Larmor frequency) reorientations of the water molecules dominate the relaxation process, and that these become increasingly affected by reducing the free water phase, finding (ii) becomes plausible; at the same time, since T_2_ is proportional to the limit of T_1_ towards zero field, this also explains the wider range of variation of T_2_ in comparison to T_1_ described in 3.1.1. (see Discussion). The quadrupole dips occur as a consequence of a crossover of the ^1^H Zeeman energy and the ^14^N quadrupolar energy levels at three separate energy levels; they become observable when protons remain in the vicinity of the ^14^N nuclei of macromolecules for a sufficient amount of time and the interaction is not averaged out by rapid isotropic motion. Reducing the water content has the double effect of increasing the relative amount of ^14^N vs. ^1^H nuclei, and of reducing the mobility of the water molecules and the macromolecules alike, so that a more pronounced interaction must be expected (iii). Comparable observations have been made earlier with aqueous solutions of glycosaminoglycans and collagen, respectively–the two main solid constituents of cartilage)–at different hydration (see below), but not for cartilage [16]. Both interpretations also explain the positive correlation that was found between cartilage relaxation dip area and severity of OA [16, 53]. For a better comparison, the contribution of the interaction with ^14^N –after subtraction of the “background” dipolar relaxation of ^1^H nuclei–is shown in Fig 6B, while the area of these corresponding peaks is compared in Fig 6C. A more than 30fold increase of the latter is found between the fully hydrated and the completely dry sample: In the dry sample, relaxation is dominated by strongly bound water molecules (possessing about 2 ms transverse relaxation time according to Table 2) whereas ^1^H nuclei in the macromolecules themselves are invisible due to the mentioned hardware limitations.

### 3.2. D2O replacement– 1.0 T

The insight obtained from the first set of experiments can be used to understand the results of the experiments involving the replacement of water molecules by deuterated water. No measurements are performed on the dry samples, only on the rehydrated ones, i.e. at approximately constant total water (D_2_O) concentration. Note the observation in 3.1.1 that T_1_ upon rehydration was found to be shorter than the equivalent value for a sample of same weight before drying; structural changes of the PG cannot be excluded and trends in T_1_ can therefore be discussed only relative to different D_2_O concentrations. However, for each type of rehydrating solution, three samples from the same joint were used in order to provide statistical evidence, and as the obtained relaxation properties were found to agree with each other within the experimental errors for all three samples used (see Table 3), the confidence in the data quality is high.

**Table 3:** T_1_ and T_2_ values of ^1^H nuclei for the two peaks identified via 2D ILT in all partially deuterated samples (description see Table 1).

Sample label	Signal	Main peak			Secondary peak		
		T_1_ (ms)	T_2_ (ms)	intensity	T_1_ (ms)	T_2_ (ms)	intensity
1Cw	451	994	96.8	439	461	0.02	12
2Cd0	807	896	82	787	672	0.02	20
3Cd0	1012	861	86.5	986	616	0.02	26
4Cd0	619	828	109	606	672	0.02	13
5Cd08	106	828	124.4	97.5	518	0.02	8.5
6Cd08	91	961	116.4	85.8	672	0.02	5.2
7Cd08	104	956	131.1	95.4	452	0.03	8.6
8Cd09	61	896	116.4	56.0	484	0.03	5.0
9Cd09	69	1116	276.8	60.0	272	0.03	9.0
10Cd09	80	1072	276.8	70.1	318	0.04	9.9
11Cd1	47	1954	479	13.8	234	0.02	33.2
12Cd1	70	1860	418	16.1	251	0.04	53.9
13Cd1	62	2413	514	3.7	331	0.03	58.3
1Tw	773	1238	157.4	761	597	0.03	12.4
2Tw	1352	1086	131	1316	514	0.03	35.6
3Td0	390	1000	59.4	379	454	0.03	10.9
4Td0	366	1000	86.3	356	452	0.02	9.9
5Td0	461	961	71.9	446	441	0.02	14.7
6Td1	48	798	290	28.1	283	0.03	19.9
7Td1	53	426	148	20.2	267	0.03	32.8
8Td1	51	530	213	22.0	297	0.02	28.9

**Fig 7:**
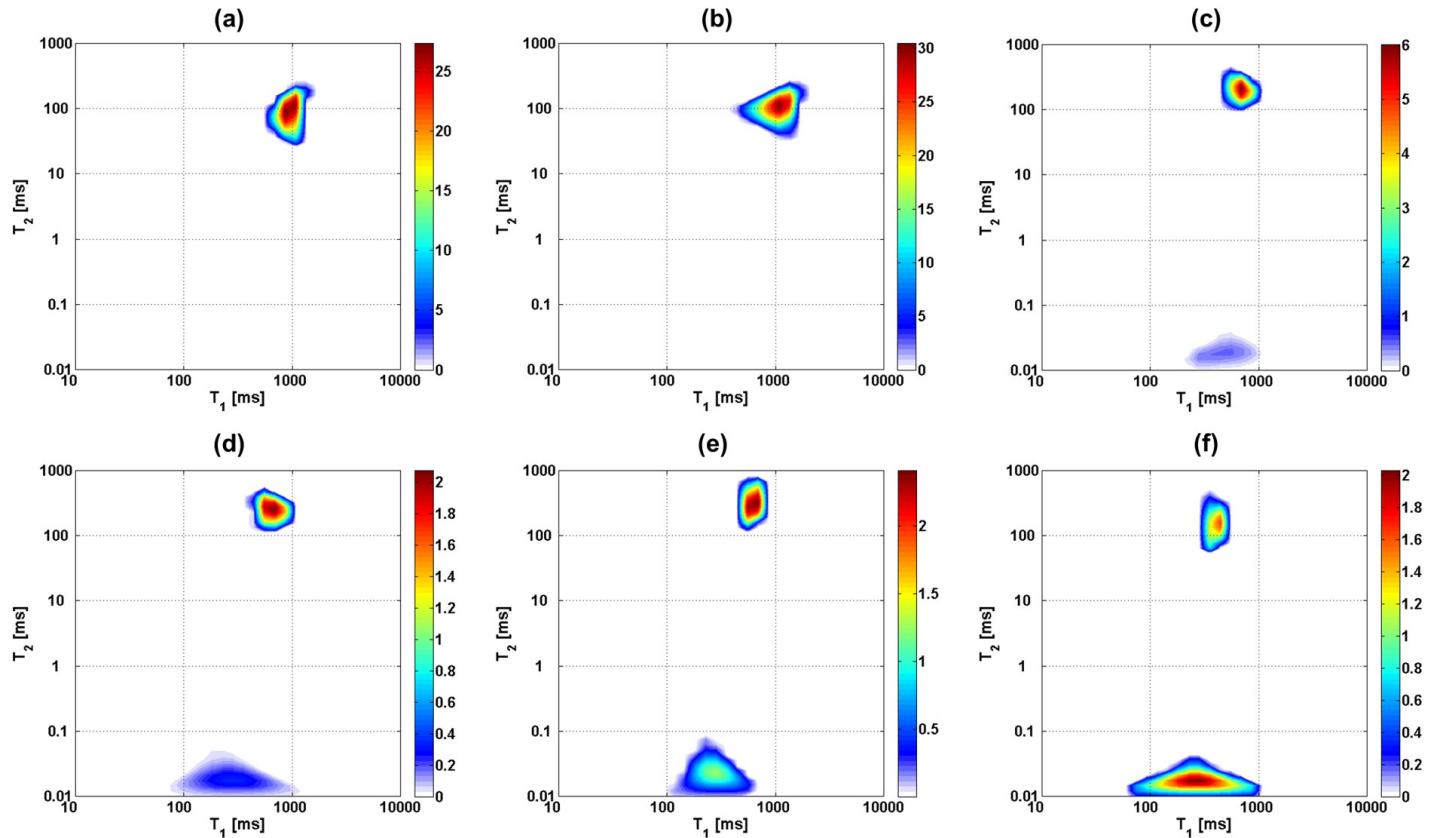
^1^H T_1_-T_2_ maps of selected cartilage samples rehydrated with different PBS solutions containing H_2_O or D_2_O: a) original sample, 1Cw, b) sample dried and rehydrated with PBS solution prepared with 100% H_2_O, 2Cd0, c) sample rehydrated with PBS solution prepared with 80% D_2_O, 5Cd08, d) sample rehydrated with PBS solution prepared with 90% D_2_O, 8Cd09, e) sample rehydrated with PBS solution prepared with 100% D_2_O, 12Cd1, f) sample after trypsin treatment, dried and rehydrated with PBS solution prepared with 100% D_2_O, 6Td1. Note that the intensity of the secondary peak increases relative to that of the main peak as less light water (H_2_O) is included in the rehydrating solution.

**Fig 8:**
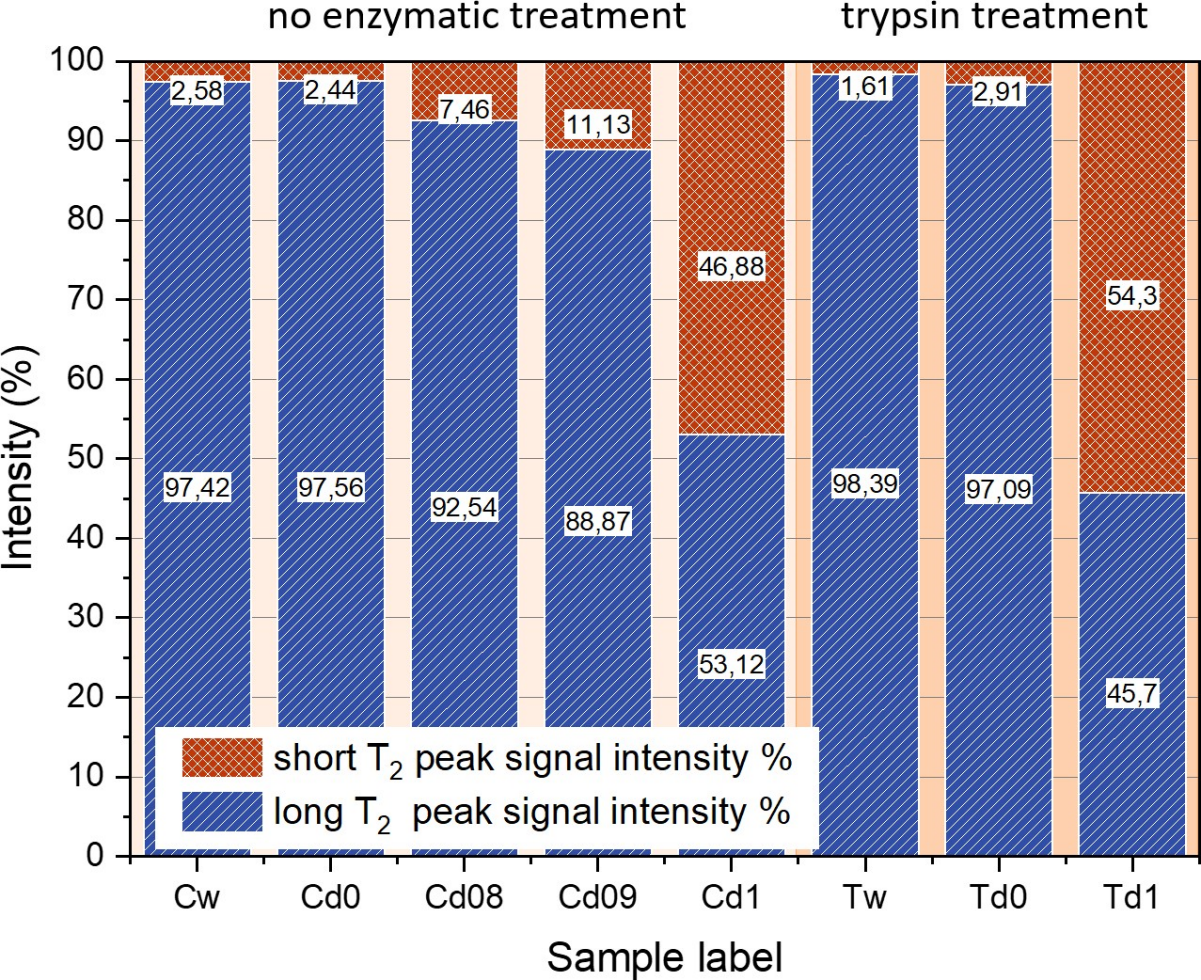
Relationship between the intensities of the two peaks identified in the ^1^H T_1_-T_2_ maps, as the sample is untreated or rehydrated with solutions with increasing concentrations of D_2_O, including the trypsin treatment.

As presented in Table 3, the first set of samples, without trypsin treatment, was used to reveal the effects of removing most of the H_2_O molecules from the sample by drying, while preserving the structure nearly intact. After the data collection via the FID-CPMG Inversion Recovery experiment and the two-dimensional Inverse Laplace Transform were performed (shown in Fig 7), the area of the main peak and the secondary peak (which becomes visible only when the concentration of D_2_O increases) have been quantified (Fig 8). The absence of the secondary peak in the 2D analysis is explained by the relatively low weight fraction of the short T_2_ peak for the untreated sample 1Cw in comparison to sample D1 in the previous part of the study (2.6% vs. 12.7%), an apparent discrepancy which is most likely due to the different age of the sacrificed animals. The ratios of the two components in T_2_, however, are well within the range of values reported in the literature which, likewise, suggests sample-dependent properties [20, 63]. While the secondary peak in the 2D plot is too close to the noise level to generate a meaningful intensity plot, it is clearly identified in the 1D time-domain datasets from which weight fractions were derived.

**Fig 9:**
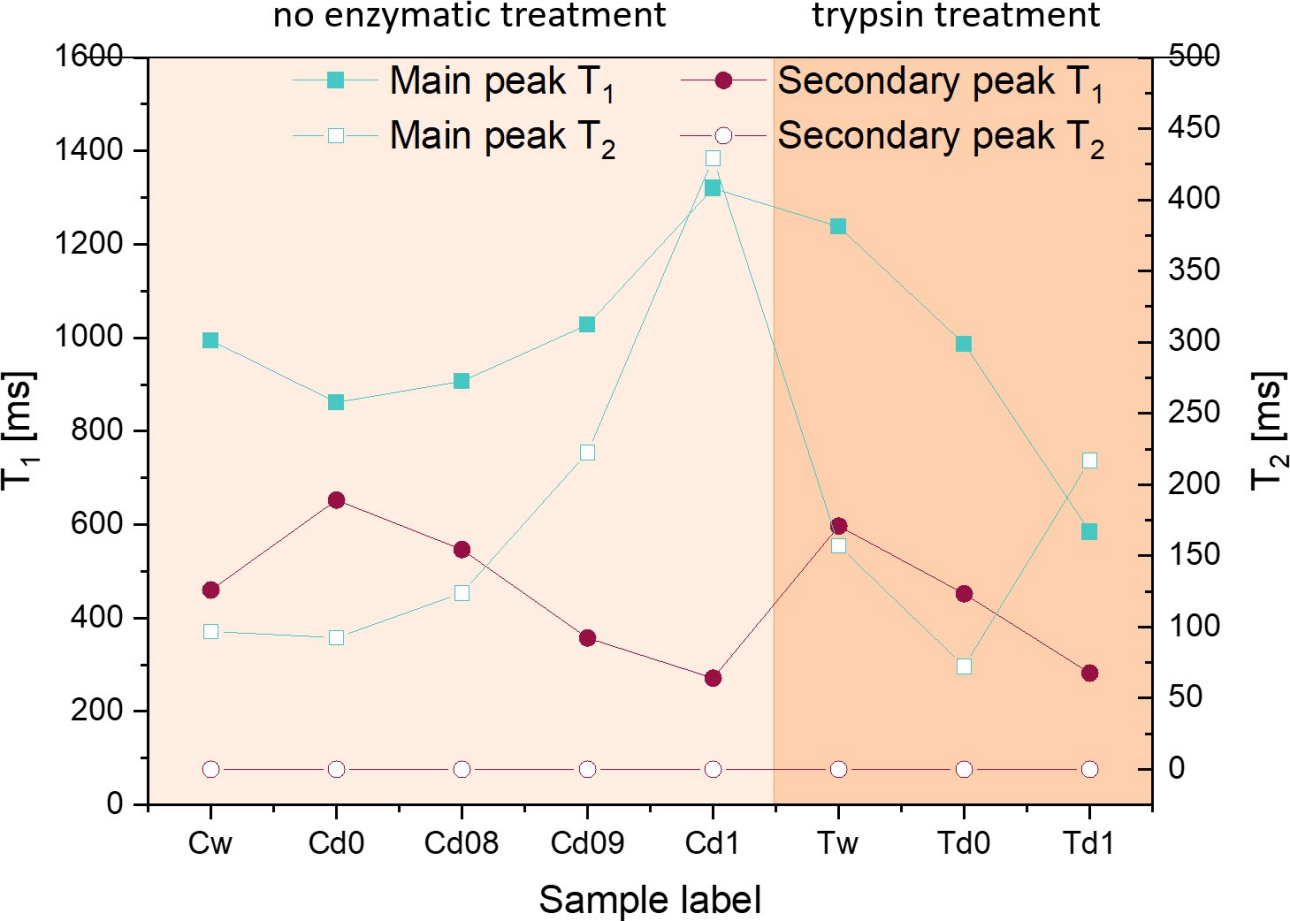
Values of the ^1^H T_1_ and T_2_ relaxation times for both peaks, for all sets of samples. Data averaged over the sets of three samples for each treatment, where available. The left Y axis represents the T_1_ values, the right Y axis represents the T_2_ values. The values of the secondary peak T_2_ remain between 20 and 40 μs.

While Fig 7 and Table 3 show results for individual samples, Figs 8 and 9 plot averages of three samples each with the same treatment. The results can be summarized as follows. Drying and rehydrating in H_2_O PBS reproduce the same relative weight fractions and also the same values for both T_1_ and T_2_ components, with a minor tendency of shorter T_1_ as seen in 3.1.2. Rehydration with a PBS of 80%/20% D_2_O/H_2_O content reduced the signal intensity by about a factor of 5, although the samples themselves were only approximately equal in size and weight so that a direct comparison of absolute signal intensities is not possible; the relative intensity, however, of the short T_2_ component peak increases by a factor of 3, and the corresponding peak becomes visible in the 2D plot of T_1_ and T_2_. For samples with rehydration in 90%/10% D_2_O/H_2_O and 100%/0% D_2_O/H_2_O PBS, respectively, this trend continues. The sample fully hydrated in D_2_O still retains about 50% signal intensity of the long T_2_ component. For that peak, both average relaxation times increase, more so for T_2_ than for T_1_. One possible explanation for this is the subsequent replacement of H_2_O molecules by a small fraction of HDO molecules due to fast hydrogen exchange between water molecules. In HDO, both the intra- and the intermolecular contribution to relaxation becomes smaller, the first due to the replacement of H by D which reduces the relaxation rate by the square of the gyromagnetic ratios, or about a factor of 42; the second due to reduction of the ^1^H spin density in the sample, i.e. by a factor of 5, 10 and infinity, respectively. This is counteracted by a slight increase of rotational and translational correlation times due to the increasing molecular mass, an effect that does not exceed 20%. Obviously, the theoretically predicted effect on T_1_ and T_2_ is much larger that the observed variation; along with the field-cycling relaxometry data, this is another proof of the fact that relaxation of water in cartilage is not dominated by the intrinsic dipolar relaxation rate which is responsible for the bulk T_1_ and T_2_ of between 2 and 3 s, but by the interaction with the macromolecular matrix, more specifically by the molecular reorientation in the presence of macromolecular surfaces (see also 3.3). This interaction also explains the short T_2_ in comparison to T_1_, whereas T_1_ = T_2_ in bulk liquids.

The short T_2_ component must have the same origin as the one in 3.1.2 for the dried samples; it consists of the signal of non-exchanging ^1^H nuclei in the macromolecules and in bound water. The majority of water protons and exchangeable hydrogen atoms such as in OH and NH have exchanged with deuterons for the sample rehydrated in pure D_2_O, but this exchange is not complete. In [19], a similar exchange process has been used and the residual ^1^H signal been analyzed. In their study, a comparable amount of ^1^H signal with long T_2_ remains. This component can only be attributed to remaining ^1^H nuclei in highly mobile water.

Note that the secondary component is characterized by a T_2_ slightly longer than that of the non-exchanged samples, and a broad T_1_ distribution, the average of which appears to be shorter than that of the first component. Unlike the situation with the dried samples in 3.1.2, this difference suggests an incomplete averaging due to magnetization transfer.

Again following a procedure analogous to that in [19], samples were treated with trypsin in order to simulate PG depletion that naturally occurs in osteoarthritis. Trypsin treatment of the samples before drying and subsequent rehydration had essentially no effect on the magnitude and the relaxation times of the short T_2_ component, despite the fact that this treatment should remove a large fraction of the proteoglycan (PG) chains, while leaving the backbone intact. The PG signal thus does not contribute significantly to the observed relaxation components. In addition, despite some scatter in the results, T_1_ and T_2_ follow the same trend upon rehydration with H_2_O and D_2_O, respectively, as found in the samples not treated in trypsin. This suggests that removal of PG also does not strongly affect the relaxation of the remaining “free” water molecules.

### 3.3. D_2_O replacement–field cycling relaxometry

Following ^1^H measurements at different stages of D_2_O replacement, the relaxation properties of the ^2^H nuclei of D_2_O itself were determined. Due to sensitivity limitations, field-cycling relaxometry was performed only for samples fully saturated with D_2_O. While water molecules with different isotopes behave very similarly with respect to chemical interaction and dynamics–save a variation of molecular motion and viscosity parameters on the order of 10–20% due to different molecular weight–, the relaxation mechanisms are not the same: relaxation of ^2^H is predominantly quadrupolar, i.e. it is determined by interactions of the nucleus with the modulations of the electric field gradient in its vicinity and is therefore strictly intramolecular; at the same time, the dipolar mechanism that dominates ^1^H relaxation has intra- and intermolecular contributions. The latter can be modulations of the interspin distance between neighboring water molecules, or with hydrogen atoms in the macromolecular matrix. Both relaxation rates, however, follow the same basic formalism (see Discussion) and are proportional to the same spectral density functions multiplied by an interaction-specific prefactor [66]. Since the intermolecular contribution to I(ω) can have several components and is generally different from the intramolecular contribution, an agreement of the frequency dependence T_1_(ω) for ^1^H and ^2^H nuclei serves as evidence that intermolecular contributions are negligible, thus facilitating the modelling of NMR relaxation dispersion in cartilage, and possibly in other tissue.

**Fig 10:**
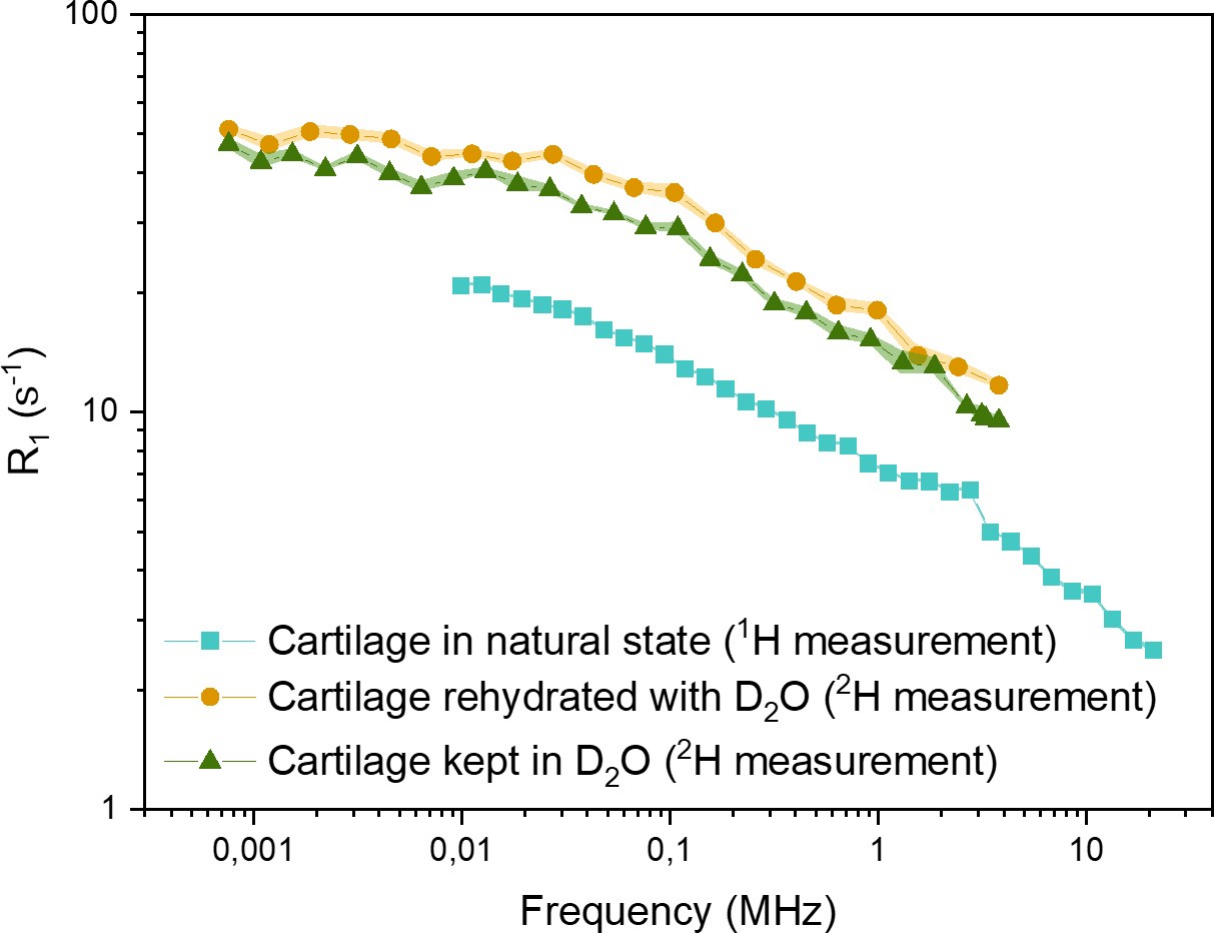
T_1_ dispersion curves of the cartilage samples kept under different conditions: Fresh cartilage (squares) - ^1^H measurements; dried cartilage rehydrated in D_2_O (circles); fresh cartilage kept in D_2_O until complete exchange, in two cycles (triangles) - ^2^H measurements. The error bars for each exponential fit are smaller than the corresponding symbol size.

For the same range of magnetic field strength at the relaxation level, about 0.2 mT to 0.5 T, the Larmor frequencies for ^2^H are shifted towards lower values by a factor of 6.5 equivalent to the gyromagnetic ratios. Where the frequencies overlap, ^1^H and ^2^H dispersions follow the same frequency dependence, so that within this range it can be concluded that intramolecular relaxation constitutes the main contribution to the total relaxation rate of water in cartilage. The results from the ^2^H measurements performed on samples dried and rehydrated with D_2_O, as shown in Fig 10, are consistent with the results from the measurements performed on samples that were kept in pure D_2_O twice for the exchange. The slight differences in R_1_ (1/T_1_) values can be explained by differing hydration states of the cartilage—the sample that has smaller R_1_ values throughout has absorbed more liquid from the environment, as it was kept in D_2_O for 48 hours. The dispersion of ^1^H of the untreated sample is shown for comparison.

## 4. Discussion

Combining relaxation times measurements at different magnetic field strengths and under different conditions aims at gaining further insight into the separate relaxation “pools” of ^1^H or ^2^H nuclei, and of the mechanism governing their relaxation. Ultimately, this knowledge can be used as a diagnostic tool to characterize tissue degradation such as in OA. In this contribution, the focus is put on low-field and variable-field devices that are increasingly becoming available for laboratory-based, but also for in vivo studies. Since the experiments cover the field range up to 1.0 T, which is not much below typical fields of clinical scanners, certain observations remain relevant also for clinical studies.

### 4.1. Identification and assignment of relaxation components

In order to model variable-field relaxation data from Fast Field Cycling (FFC) experiments, its current limitations need to be considered: (a) signal acquisition often involves detection by means of CPMG trains, so that the short T_2_ component becomes essentially invisible; (b) whole cartilage sections are studied so that the measured result represents an average of T_1_ across the whole tissue; (c) measurements of ^2^H are, with existing hardware, only feasible with fully hydrated samples. Limitation (a) means that the measured T_1_ corresponds to the long T_2_ component that was identified in the experiments carried out at 1.0 T. Considering (b), while the width of the T_1_ distribution for the mentioned peak is comparatively narrow at 1.0 T–never exceeding half an order of magnitude, see Figs 2 and 7 –, it was found that this width probably increases at intermediate or low Larmor frequencies, see [17, 50]. As a working hypothesis, we assume that the frequency dependence of T_1_ is qualitatively given by its average value, as only trends and relative changes are discussed.

The observed relaxation time of a spin system is determined by the intrinsic mechanism responsible for molecular relaxation itself, and the type of coupling that exists between different pools of spins. For the second aspect, detailed studies have been carried out with cartilage and collagen at varying hydration levels, and an attempt was made to explain concentration- and time-dependence of relaxation rates in bovine cartilage tissue by a coupled system involving free (bulk) water, proteoglycans, collagen and collagen-fibrillar water (CFW) [19, 20]. In that work, exchange rates between free water and the remaining phases were observed to be between 0.2 s^-1^ (collagen) and 0.7 s^-1^ (PG, CFW). We assume that, with all components and environmental conditions such as temperature and pH remaining similar, these numbers represent good approximations for any kind of cartilage tissue. This observation then suggests that, for the relaxation values obtained in this study, exchange is between the *slow* and the *intermediate* regime. Note that, as T_1_ becomes much shorter towards lower magnetic field strength, exchange effectively becomes *slow* under these conditions, so that multiexponential relaxation can be expected, and the T_1_(ω) measured can be assigned to the bulk water phase exclusively if the remaining three phases do not contribute due to their short T_2_. Assuming that the above exchange rates also describe the bovine cartilage samples under study, *slow* exchange can be concluded for the entire range of Larmor frequencies in the FFC experiments where the minimum observed relaxation rate was found to be about 2.5 s^-1^.

The situation is somewhat different for the 1.0 T experiments where relaxation times are closer to 1 s; the similarity of both T_1_ components may indeed be a coincidence, but it can also be a consequence of exchange processes either of magnetization, or–in a physical sense–of hydrogen nuclei or water molecules. The ^1^H data of partially deuterated samples suggest that, if exchange takes place, it must be *intermediate*. Furthermore it is reasonable to assume that deuteration does not affect the short-T_2_ component, despite it potentially containing a fraction of structural water molecules; although the quantification of the short-T_2_ component is not entirely reliable, its T_1_ range remains unaltered for all dehydrated and D_2_O-diluted samples. It is therefore reasonable to conclude that the long-T_2_ contribution truly describes the relaxation properties of the “free” water phase, in experiments at all frequencies in this study. Note that the same may not be true for high-field studies where T_1_^-1^ can assume values on the order of the exchange rates.

It appears appropriate to mention that the two-dimensional representations of Figs 2 and 7 are plots of correlation, not exchange, since they lack a mixing period. They thus convey a somewhat different information as that provided in [19, 20] but qualitatively agree in terms of relaxation times values. Actual two-dimensional exchange experiments on tissue samples were presented for ^2^H nuclei in [67, 68]. The first such correlation experiments were presented, to the best of our knowledge, by Mailhiot et al. [31] for severely diseased human tissue, but at a magnetic field strength of 5.78 T. In that work, the short T_2_ component was outside the experimental detection window but a second component was indeed observed and has been assigned to biopolymers with a comparatively high mobility, as was proven by diffusion measurements [69]. This component was neither observed, nor indeed expected, in our study of *healthy* bovine tissue. In fact, separate diffusion experiments were performed on the untreated cartilage samples of this study in the Spinsolve instrument to confirm that the long component is composed of bulk-like water molecules that are embedded in the matrix, but have no direct bonds with the solid matrix. The measured diffusion coefficient is about 50% the value for free water molecules in bulk, which is explained by the confining nature of the cartilage structure, even when fully hydrated. This value represents an average across the tissue structure and is in good agreement with published results of diffusion coefficients in cartilage [35, 70].

In general, as was mentioned in the Introduction, apparently conflicting results have been discussed in the literature over the last decades, where either one or two or even three transverse relaxation components were identified. The reasons for this observation are discussed in [1], Chapter 18, but there seems to be no definite answer–apart from obvious differences between the cartilage composition in different mammals, the magnetic field dependence and the related relative value of the water exchange times can be held responsible for some of these differences. First of all, the very short component below 100 μs, as presented in this work, is often neglected since it is considered irrelevant for clinical and preclinical studies because it is either invisible to the hardware, or can easily be filtered out. The remaining water relaxation times T_2_ are distributed both due to structural heterogeneities on a macroscopic (layer structure inside cartilage) and microscopic level, and by the well-known orientation dependence of T_2_, i.e. water among fibers with a broad distribution of angles with respect to the B_0_ field will automatically lead to an accordingly broad distribution function P(T_2_) [71]. Averaging between these individual values, on the one hand, again depends on the actual values of T_2_ and on the structural sizes. On the other hand, it depends on the mathematical effort used for separating similar components–without proof, one may state that a broad distribution covering a factor of 10 will often split into two or even three components, depending on the algorithm used, while relaxation times covering a factor of two will be interpreted as a monoexponential function by most fitting programs, and indeed by the human eye. Commercial ILT codes, such as the one used in this study, either identify components that are separate from each other or integrate them into a single peak with a certain width. In this study, in no case could a separate relaxation time contribution outside of the discussed two fractions be identified. This has been verified with a wide range of regularization parameters and also using the Butler-Reed-Dawson method [22]; it was found that any additionally occurring peaks were not robust and reproducible, and were therefore considered spurious by us. We are therefore certain that no peak in excess of 1–2% weight exists in the samples under study. As mentioned above, this does not represent a contradiction with reported larger numbers of peaks found in different tissue and with different equipment.

Following the above argumentation, having ensured that no significant physical or magnetization exchange occurs in the described system, and that the observed long-T_2_ contribution can be attributed to free water in its entirety, it is now possible to address the actual mechanisms that govern relaxation of this water phase.

### 4.2. Short and long transverse relaxation time component

Let us first discuss the component of short T_2_. This peak must be a contribution that is not in physical exchange with the other component, at least not on a timescale of the longitudinal relaxation times, i.e. about 0.1–1 s. This does not exclude the presence of magnetization exchange, which has been investigated by time-domain studies, in particular in [19]. Its apparent T_2_ value remains constant between 20 and 30 μs, it is characterized by a Gaussian decay typical for strongly dipolarly coupled spins. The absolute intensity of the second peak remains rather constant at a value that is equivalent to about 14% at full hydration. A direct comparison with the actual weight is, however, only possible if the proton density of macromolecules is compared to that of water. Considering 2/18 for water and about 40/640 (the value of heparin sulfate) for PG, the 14% contribution to the signal is equivalent to about 23 wt-% of PG and 77 wt-% of water, a value closer to the determined weight ratio. Despite extended discussion in the literature, different results were found for the actual amount of “bound” water with a very short T_2_, however, it can be safely assumed that the procedure used in this study leaves a certain amount of residual water in the sample, and the consistent result that all ILT analyses render only two distinct relaxation components suggests that the peak with short T_2_ contains protons in macromolecules as well as in water, while this peak remains unresolved. Note further that the T_1_ values of the secondary peak apparently possess a broader distribution than that of the primary peak and are slightly shorter, but that the mean value follows the same tendency on dehydration so that, within experimental error, both peaks are characterized by the same average T_1_. The shorter T_1_ in the secondary peak of the D_2_O-exchanged samples may be attributed to the fact that it stems from water molecules and biopolymer protons not fully exchanged, while the “free” primary component essentially originates from HDO molecules with their longer intrinsic relaxation time compared to H_2_O. Indeed, increasing deuteration from 0% through 80% and 90% to ~100% leads to progressively shorter average T_1_ of the secondary component, as would be expected under these assumptions. The approximate agreement between T_1_ of primary and secondary peaks may indeed suggest magnetization exchange faster than the typical timescale for T_1_ of 100 to 1000 ms. This finding agrees with the more detailed study [19] who found additional, but small, signal contributions but determined T_2_ from the FID rather than from CPMG trains. In addition, our observations agree well with the finding of spatially resolved ^1^H T_1_ in D_2_O exchanged cartilage by Tadimalla et al. [56] where a variation along the tissue depth was found, this can explain the volume-averaged T_2_ distribution of the secondary peak which was observed to show a broader distribution when compared to the non-exchanged samples. The same was not observed by [56] for T_1_, but this would be expected for experiments carried out at 7 T where T_1_ variations are small, while they are expected to be larger at 1.0 T used in this study (compare secondary peak in Figs 2 and 7).

The main interest for most studies, in particular in the biomedical context, is certainly on the component of long T_2_. We here assume the notion of a porous medium where a fluid interacts with interfaces, the latter representing relaxation sinks by different types of coupling (see below). With the assumption of water molecules essentially being freely mobile, a given fraction p_a_ would be considered “adsorbed” at any time, being in *fast exchange* with the remaining fraction (1-p_a_) which behaves bulk-like. Assuming a residence time very short compared to T_1_ and T_2_, fast exchange is observed for these fractions if the geometric dimensions allow the molecules to reach the closest interface several times during the relaxation time–given the self-diffusion coefficient of water, this requirement is certainly met for the biological structural sizes well below 1 μm. (Note that this timescale is different to the longer ones discussed above which relate to the physically different fraction of protons of bound water or macromolecules). The dependence of relaxation times on water content is in full agreement with observations made in porous media where relaxation is a weighted average between a bulk component and an efficient surface relaxivity; in this simplified consequence of the Brownstein-Tarr derivation [72], the observed relaxation rate 1/T_i_ is proportional to the surface-to-volume ratio, or inversely proportional to the amount of free water:

$${1/\text{T}}_{\text{i}}{=\text{p}}_{\text{a}}/\left({\text{T}}_{\text{i},\text{surf}}\right)+\left({1-\text{p}}_{\text{a}}\right){/\text{T}}_{\text{i},\text{bulk}}{\sim \text{S}/\text{V}*\rho }_{\text{i}}{+1/\text{T}}_{\text{i},\text{bulk}}$$ (1)

where S and V are surface and volume of the pore structure–in this case, these can be approximated by the surface of PG and collagen fibers, and by the remaining fluid volume–, _i_ is the surface relaxivity in units of μm/s, and i = 1,2.

**Fig 11:**
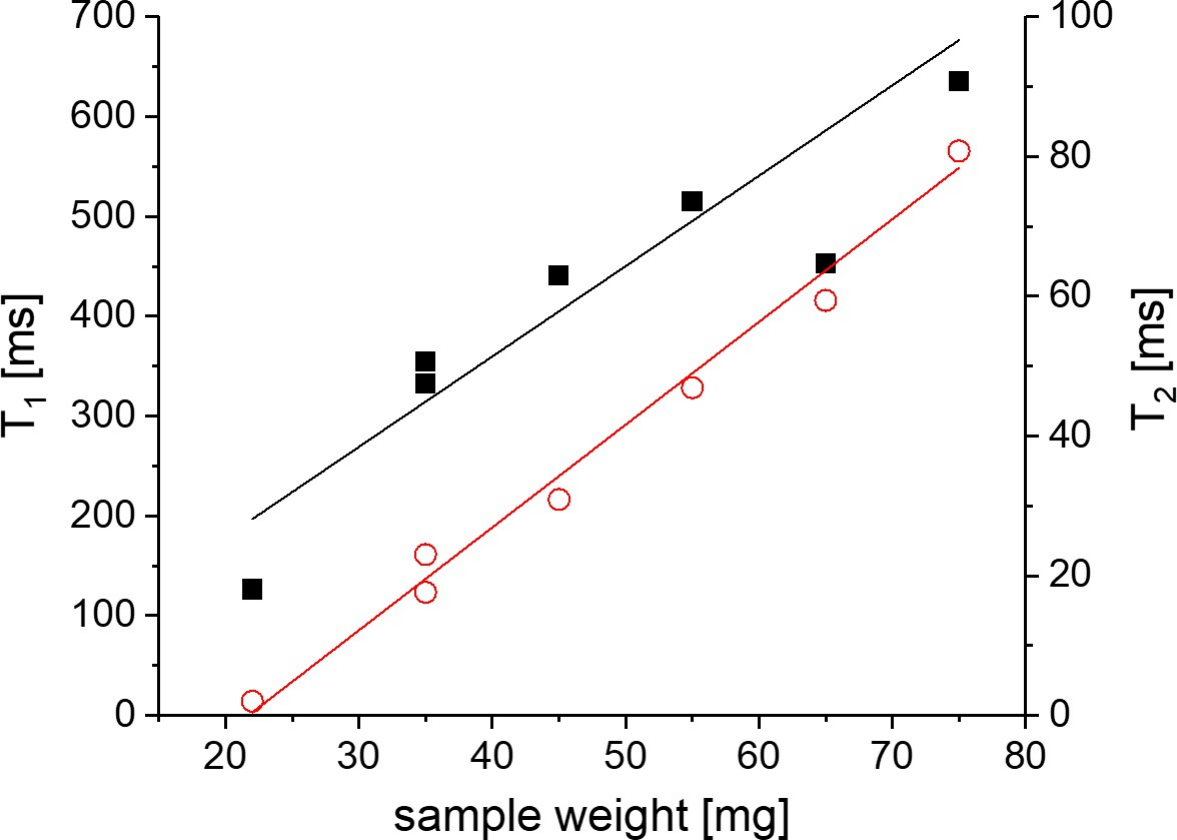
Dependence of relaxation times T_1_ (black solid symbols) and T_2_ (red open symbols) at a magnetic field strength of 1.0 T for a partially dried cartilage sample (labeled as D1 to D5) as a function of sample weight. Solid lines are the best fits to linear dependences.

As can be seen from Fig 11, ρ the assumed proportionality is well observed for T_2_, and to a lesser degree also for T_1_. The deviation can possibly be explained by the absence of a sufficient amount of “free” water in the dry samples, prohibiting fast exchange according to Eq 1. [20] suggests that “bulk-like” water exists only for a mass ratio H_2_O/dry mass above 1.57, whereas water associated with proteoglycans represents the dominant mobile phase for mass ratios between 0.37 and 1.57. These limits correspond to water weight fractions of 27% and 61%, respectively. Considering that the drying process in [20] was similar to the one carried out in this study, it appears that only the completely dried samples do not possess water fractions that are either free or PG-associated, so that the remaining water phase remains spatially hindered, a possible explanation also for the deviating relaxation dispersion behavior (see below). In a similar study on concentration dependence, Damion et al. [73] suggest to consider the change of macromolecular mobility at low water concentration as a further influence that will render T_1_^-1^ and T_2_^-1^ not strictly proportional to the inverse water concentration.

### 4.3. Modelling the frequency-dependence of longitudinal relaxation times

In discussing Eq (1) it needs to be kept in mind that all relaxation terms are frequency dependent. In this derivation, it is assumed that water remains under fast exchange conditions in all cases, and that the surface-to-volume ratio of the cartilage sample remains unchanged also upon drying, a statement that can only approximately be true. Nevertheless, the network of PG and collagen fibers assumes the role of a relaxation sink, i.e. the interior surface of a porous medium. Due to the fact that T_2_ is more sensitive to slowly modulated magnetic fields (i.e. slow molecular motion), the effective surface relaxivity ρ_2_ is usually larger than the corresponding value for T_1_, ρ_1_, unless very low Larmor frequencies are considered. From observations of all studies of water dynamics in a wide range of porous media, organic or inorganic, one finds that there is substantial contribution to the relaxation dynamics at frequencies of 1 MHz and below, so that the studied field of 1 T (corresponding to 43 MHz ^1^H Larmor frequency) can already be considered rather “high”. This is equivalent to the description of the Brownstein-Tarr equation assuming that the actual T_1_ and T_2_ approach their bulk value which is about 2 s for the brine solution encountered in cartilage. The results are thus in agreement with the two-site/fast exchange model, without specifying the exact nature of the surface relaxivity. At the same time, and again of general validity, are the functions that determine the frequency dependence of dipolar as well as quadrupolar relaxation with respect to the Larmor frequency ω: [66]

$${1/\text{T}}_{1}\;(\omega )=\text{const}\;[\text{I}(\omega )+4\text{I}(2\omega )]$$ (2)

$${1/\text{T}}_{2}\;(\omega )=\text{const}/2\;[3\text{I}\left(0\right)+5\text{I}(\omega )+2\text{I}(2\omega )]$$ (3)

with the spectral density function I(ω) and const = (μ_0_/4π)^2^ γ^4^ ħ^2^/5 r^-6^ I (I+1) for homonuclear dipolar relaxation, and const = 3/80 (e^2^qQ/ħ)^2^ (1+ η^2^/3) for quadrupolar relaxation, respectively, where γ is the gyromagnetic ratio, *I* the spin quantum number, *r* the internuclear distance, *Q* the quadrupolar moment, *q* the electric field gradient and *η* the asymmetry parameter of the electric field. Note that the formal structure in terms of spectral density function is the same for both relaxation mechanisms.

From these equations one can make the common approximation T_2_(ω) ≈ 10/3 T_1_(0)—or, more generally, T_2_ at any magnetic field strength is proportional to the zero-field limit of T_1_, which can often be estimated if the dispersion approaches a plateau at low fields. The ratio T_1_/T_2_ therefore gives an indication of the strength of the frequency dependence of T_1_, or dispersion.

Relaxation occurs by reorientations of the spin-bearing molecules, leading to magnetic field fluctuations experienced by the spins themselves, or by coupling to neighboring spins that is modulated by relative motion. The spectral density I(ω) is the Fourier transform of the autocorrelation function of orientation, G(t), and can be computed if a suitable model for the latter is available. Apart from the intrinsic relaxation mechanism of the bulk fluid, which is readily understood and provides frequency-independent relaxation times T_1_ = T_2_ due to the short molecular correlation times τ involved (ωτ << 1), several mechanisms are considered that may contribute to the observed relaxation dispersion T_1_(ω) and are known from different liquid/solid interface scenarios: (a) interactions with unpaired electrons or paramagnetic centers; (b) direct relaxation with nuclei (e.g. protons) in the biopolymer matrix; (c) indirect frequency dependence via magnetization transfer from the biopolymer matrix, thereby “copying” the relaxation dispersion of the latter, being linked with the biopolymer dynamics; (d) interaction with specific sites, for instance via hydrogen bonds, allowing for characteristic residence times of the water molecules; (e) reorientations mediated by translational displacements, or RMTD. All five of them would generally provide relaxation rates of the “free” phase in proportion to the water content according to Eq (1), under the assumption that the biopolymer maintains its structure and dynamics irrespective of hydration level.

For discrimination between these contributions, the results of the ^1^H and ^2^H relaxation dispersion experiments (Fig 10) are of vital importance:

- is inapplicable due to the very low concentration of paramagnetic centers in the tissue, but would be dominating in many inorganic solids such as rocks;
- can be ruled out from the D_2_O measurements since they would be inefficient due to the small dipolar coupling of ^2^H and other nuclei–the dipolar contribution to ^2^H relaxation is typically two to three orders of magnitude smaller than the quadrupolar contribution, so that the latter dominates in most cases with the possible exception of large unpaired electron densities leading to ^2^H-electron dipolar coupling. Case (b) is indeed relevant for ^1^H coupling with ^14^N nuclei, giving rise to the pronounced peaks in relaxivity at the crossover of ^1^H Zeeman energy and ^14^N quadrupolar energy levels;
- is possibly effective towards higher fields, and is expected to be much smaller for ^2^H;
- has already been suggested for cross-linked bovine serum albumin by Koenig and Brown [44] but again leads to different results for ^1^H and ^2^H;
- is considered here as the main contribution, in particular with reference to the similarity of dispersion shown in Fig 10.

The RMTD mechanism suggests that molecules undergo reorientations not only by fast rotation and translation, but also by being forced in geometrical restrictions. This requires them to assume preferential rather than random orientations relative to a surface, for instance by interaction due to permanent or induced electric dipoles. In fact it has been shown with inorganic glasses that polar molecules on polar surfaces follow RMTD while non-polar molecules generally do not [49]. Under these conditions, the orientational autocorrelation function G(t) does not decay to zero by molecular rotation, but assumes a non-zero limit which only decays when the molecule experiences different surface orientations by means of translational diffusion, taking place on a longer timescale. RMTD does not require long interaction times of the molecule with the surface and therefore does not significantly slow down translational diffusion itself; unlike all other contributions mentioned above, it represents a single-particle motion and excludes intermolecular interactions. This was first demonstrated by the identity of spectral density functions of water ^1^H and ^2^H relaxation in a porous glass [49]: since I(ω) of the quadrupolar nucleus ^2^H is intramolecular in principle, it must be concluded that a possible intermolecular contribution to relaxation of ^1^H in water, which would be of a different nature and follow a different frequency dependence, must be negligible. The comparison of ^1^H and ^2^H relaxation dispersion shown in Fig 10 is the first experiment on biological tissue that confirms a similar situation for water in cartilage. While this finding is in agreement with the suggestion of water reorienting on the surface of the collagen network made in Tadimalla et al. [56]–where we find that interaction with both collagen *and* GAG may be relevant–, we do not find the necessity of assuming intermolecular interaction with protein protons since this would not lead to the observed ^2^H relaxation properties.

A connection to the surface structure has been attempted by introducing a distribution function S(*k*) of modes *k* which are experienced by the molecule, assuming that a totally flat surface would not lead to any molecular reorientation, while pore diameters or surface roughness parameters on a scale exceeding the molecular size are suitable to affect relaxation via RMTD. S(*k*) can be approximated to be a constant in between cutoff values, but it is more likely related to the actual distribution of curvatures in a real sample, and a power-law distribution S(*k*) ~ *k*^*-χ*^ has been assumed [74, 75] since it can be translated into a power-law of the frequency dependence of T_1_, T_1_(ω)~ω^γ^, which is often observed. χ is a parameter that has been linked to the surface “roughness” vie the Hurst exponent [76] where 0 < χ < 1. The relation between χ and γ depends on the type of diffusion with respect to the sampled surface: for normal diffusion with a Gaussian propagator one finds γ = (1 + χ)/2, while for Cauchy statistics, i.e. molecules carrying out Lévy walks along the surface, γ = χ [49, 76]. Apart from liquids in porous glasses [49, 77, 78], on silica fine particles [74] and on clay platelets [79], the RMTD formalism has, for instance, been applied to anomalous diffusion in eye lenses [80] and to organic molecular gels [81, 82] and cellulose gels [83], respectively, where normal diffusion has been assumed. Gel concentrations in those studies ranging from 0.5 to 4% are even lower than the total amount of collagen and proteoglycans in cartilage which is on the order of 15–25%; nevertheless, subtraction of the bulk T_1_ contribution resulted in RMTD-related power-law exponents γ between 0.3 and 0.55 in those works.

Cross-linked gels have repeatedly been described as suitable systems for modelling water behavior in tissue [44], and they appear to be a particularly good concept for modelling water dynamics in cartilage which has no further geometric hindrances to water motion than the collagen fiber network and the intertwined proteoglycan network, neglecting the 1–2% of chondrocytes. We thus assume relaxation due to RMTD processes of water interacting with these networks as a working hypothesis for the origin of T_1_ dispersion in cartilage. We hereby propose a modified RMTD process; whereas in pure RMTD, the nature of the interaction of the liquid molecule with the surface is not specified and of short-term nature, hydrogen bonding and possibly further geometrical restrictions may be encountered by the water molecule in presence of PG and collagen. The purpose of this assumption is to reproduce the observed properties and to suggest an interpretation of earlier studies on cartilage and its constituents, while not attempting a quantitative analysis in terms of structural properties. Note that protein protons, although they do possess a distinct T_1_ dispersion by themselves [84, 85], do not contribute to the signal in our field-cycling experiments; the dispersion of water protons is considered to be different due to the slow exchange process [86].

The exponent γ = 0.27±0.02 describes the T_1_ dispersion T_1_(ω)~ω^γ^ in the region below the occurrence of the quadrupolar peaks (see Fig 6) down to frequencies of several 10 kHz. This exponent found for bovine tissue in this study is in perfect agreement with human articular cartilage studied in [55] where it was found that the exponent of γ = 0.26 remained constant irrespective of severity of osteoarthritis, while the exponent in the upper frequency range, i.e. above about 1 MHz, is consistently larger and correlates with the degree of osteoarthritis expressed in Mankin grade, though this correlation was at the limit of statistical significance in that study.

^2^H relaxation dispersion in Fig 10 follows the same frequency dependence where the frequency range overlaps with that of the ^1^H measurements. This confirms the dominance of intramolecular, i.e. rotational, contributions to relaxation for water molecules in the discussed frequency range. Due to the gyromagnetic ratio of ^2^H being 6.5 times smaller than that of ^1^H, the accessible Larmor frequency range is shifted by a similar amount, revealing a tendency towards flatter dispersion of about γ = 0.1 below 10 kHz. Note that this ^2^H T_1_ behavior is in qualitative agreement with ^1^H T_1ρ_ results obtained at different lock field frequency ω_L_ in the kHz range [87], [1] chapter 5, as might be expected from the similarity of the expression for the relaxation rate, 1/T_1ρ_ (ω_L_) = const/2 [3I(ω_L_) + 5I(ω) + 2I(2ω)]; however, unlike T_1_, T_1ρ_ is known to be strongly orientation-dependent [64, 88, 89] so that a direct and quantitative comparison between the dispersions T_1_(ω) and T_1ρ_ (ω_L_) is not possible.

It should be mentioned that, while deuteron relaxation dispersion data for biotissue are not available, early works by Kimmich et al. [84, 85] have treated the T_1_ dispersion of ^2^H nuclei of the hydration layer in lyophilized proteins such as bovine serum albumin in great detail. Surface diffusion with simultaneous reorientation is similar to the case in this study, but the large macromolecular complexes in cartilage do not tumble and probably do not show dynamics beyond small-scale oscillations; at the same time, the distribution of reorientational modes *k* is generally not assumed to be a step function, but may be expressed in terms of a distribution function reflecting the microstructure of collagen and the glycosaminoglycans (GAG), respectively. The power-law S(k)~k^-χ^ mentioned above is a mere viable approximation for describing the experimental data.

In the dried sample DF3, the T_1_ dispersion for ^1^H nuclei is more pronounced, as can be seen in Fig 6 –the exponent assumes the value of γ = 0.45. Although the apparent exponent is given by the discussed fast-exchange averaging discussed earlier, i.e. 1/T_1_(ω) ~ S/V *ρ_1_(ω) + 1/T_1,bulk_, where 1/T_1,bulk_ is frequency-independent and about 0.5 s^-1^, averaging does not significantly affect the values of 1/T_1_(ω) below Larmor frequencies of 1 MHz where 1/T_1_(ω) exceeds 10 s^-1^. The difference in slope must therefore be attributed to deviations of water distribution and reorientation dynamics. This naturally raises the question whether the observed T_1_ dispersion is dominated by the proteoglycan or the collagen part of the system. In earlier studies, it was concluded that both biopolymers contribute to the presence of ^14^N relaxation dips, since both contain a comparable amount of ^14^N nuclei; a semi-quantitative discussion of the influence on dispersion has not been made, but the fact that below 1 MHz Larmor frequency for ^1^H nuclei, T_1_(ω)~ω^0.27^ was maintained for different degrees of osteoarthritis in the human study [55], where PG content was expected to vary significantly, suggests that a mere reduction of PG does not affect dispersion in an obvious way. In the dried sample DF3, the swelling properties of water are much reduced and the structural integrity of the PG network is expected to be affected, altering the mobility of water molecules, while water-collagen interaction may not be changed in the same degree. The increase of the exponent γ from 0.27 to 0.45 must therefore be assigned to the variation in PG structure.

Further evidence comes from earlier comparative studies of water in collagen and GAG suspensions, respectively [16]. While the materials are certainly not identical to human or bovine GAG and collagen, this study suggests, on the one hand, a slight decrease of γ after trypsin treatment whereas the enzyme collagenase, partially destroying the collagen network integrity, does not have the same effect. On the other hand, dispersion of water protons in fully hydrated GAG and collagen suspension was found to be almost exactly the same at γ = 0.26, but drying has a more pronounced effect on water in the GAG suspension. A thorough separate study of either GAG or collagen networks seems unlikely to render precise dynamic information since it is not expected that a microstructure identical to the real situation in mammalian cartilage can be reproduced artificially. We therefore conclude that the T_1_ dispersion in native tissue is governed by interaction of water with the biopolymer components by means of the RMTD process, with interactions to both components being indistinguishable under biologically relevant water concentrations, possibly further supported by the fast exchange of water between the collagen and PG moieties in close proximity. Only at unphysiological states of drying, the T_1_ dispersion below Larmor frequencies of 1 MHz becomes more pronounced, an observation more strongly affected by the eventual collapse of the swollen PG network.

### 4.4. Relaxation times in dehydrated and rehydrated samples

Finally, a tentative explanation will be attempted for the trypsin-treated cartilage. The discussion is based on the comparable sets Cw/Tw, Cd0/Td0, Cd1/Td1, where each label represents an average over three separate samples (see Table 3). A direct comparison to the study of Lattanzio et al. [19] is not possible because they report reconstructed FID decay times rather than T_2_ times measured by CPMG, but a slight increase of both visible T_1_ and T_2_ components is observed in both studies, here corresponding to samples Cw/Tw. Although the amount of removed PG is not known, we assume a loss of between 40% and 60% based on earlier experiments [16]. Reduction in the amount of relaxation sinks, equivalent to a smaller “surface” term in the derivation of Eq 1, explains the observation that T_1_ and T_2_ become closer to their bulk values in the case of fast exchange.

However, it is less straightforward to explain the variation for the rehydrated sample pairs Cd0/Td0 and Cd1/Td1. On the one hand, the secondary component follows a similar trend with and without trypsin treatment, i.e. the effect on T_1_ is rather small and the relative proportion of the secondary peak increases from 47 to 54%—a counterintuitive observation if one assumes that this peak originates from bound water and a fraction of biopolymer protons, both being reduced after PG depletion. Nevertheless, this trend can be expected from the frequently observed change of free water fraction upon rehydration which was mentioned earlier. On the other hand, the primary component, which should follow the trend of longer T_1_ due to removal of relaxation sites, actually shows a shortening of T_1_ and an intermediate behavior for T_2_, becoming consistently shorter for H_2_O-rehydrated samples but longer for D_2_O-rehydrated samples, each trend being confirmed for all three separate samples.

We are not aware of any previous study on comparable dehydration/rehydration cycles. Under in vivo conditions, the local water concentration inside the joint varies significantly under mechanical load, and relaxes within seconds upon removal of that load, supported by the osmotic potential of the GAG. One way to demonstrate this *ex-vivo* has been osmotic pressure investigations, predominantly studying NMR spectroscopic properties [90, 91]. In the excised sample, however, this overall balance is disturbed since the joint capsule is not maintaining the total water balance any longer; therefore, excised cartilage tissue experiences superhydration and corresponding swelling. While the weight of the sample can be controlled and can be kept deliberately constant, the actual water distribution inside the tissue may change. In this study, samples underwent drying/rehydration cycles of identical timing and external conditions, and it is assumed that this will render comparable water distributions practically independent of the H_2_O/D_2_O ratio; however, it cannot be concluded that these distributions are equal to the freshly excised or untreated sample, so that relaxation times and weights of the rehydrated samples may be affected.

One possible explanation may be the eventual removal of mobile PG still in the sample and the consequential collapse of the remaining PG network, while the collagen fibers remain unaffected–water molecules will then have a higher probability to come into contact with collagen since the buffering effect of the charged and swollen PG network is less prominent, and a higher surface relaxivity can be assumed for collagen in comparison to PG for the same mass ratio, as is indicated in the results found for GAG and collagen suspensions of different water content in [16]. This can indeed explain the trend found in Fig 9 for Td0. The role of deuteration must then be assigned in a similar fashion as the trend found for the non-trypsinated samples, i.e. an increase of T_2_ from Td0 to Td1 because the remaining molecules are HDO where intramolecular relaxation is essentially vanishing.

## 5. Conclusions

NMR relaxation of nuclei in bovine articular cartilage has been studied by means of ^1^H and ^2^H relaxometry, addressing variation of water content, dilution with D_2_O and treatment with trypsin. For the first time, a systematic study of the correlation of relaxation times T_1_ and T_2_ under these varying conditions was presented, and the solid-like component has been quantified by means of a pulse sequence combining FID and echoes. Likewise, this represents the first study of deuteron relaxation dispersion measurements in cartilage.

At a magnetic field strength of 1 T, which is considered low in comparison to many laboratory fields but quite comparable to typical field strengths in many standard clinical MRI machines, healthy bovine articular cartilage is characterized by two distinct relaxation components with very different T_2_ but similar T_1_ values. Both components are rather narrow in distribution, i.e. between one half and one order of magnitude, in agreement with earlier observations that the total range of T_2_ across many unoriented articular cartilage samples is on the order of 5 while T_1_ covers a somewhat wider range at low field strength below 0.1 T, but shows rather small variation at 1 T and above. Other components, as have been reported occasionally in the literature, are not observed above a detection limit of about 1%. These components were connected to mobile biopolymers, to a spread of orientation-dependent T_2_ in certain sample geometries, or possibly to sample-specific variability.

The “primary” component, being identical with the one that is acquired in imaging experiments, consists of the signal of bulk-like water molecules that are mobile between the superstructure of the approximately 25% proteins, i.e. the intertwined proteoglycan and collagen networks that stabilize the cartilage frame. This component is considered as a fluid interacting in a porous medium with high permeability, with the translational diffusion coefficient about half of that of free water and therefore short interaction times with a protein scaffold that is essentially immobile, save for small-scale fluctuations. The pronounced dependence of T_1_ on Larmor frequency, the so-called dispersion, can be analyzed in analogy to liquids moving in the pore space of an inorganic porous medium. Both the T_1_ and T_2_ values at 1T as well as the dispersion follow the behavior expected from fast exchange conditions between two relaxation pools, i.e. free water and surface water; spatially resolved measurements with the NMR-MOUSE show that drying occurs homogeneously across the whole tissue. All experimental results agree with earlier studies that a definite assignment of water relaxation to interaction to either the collagen or the proteoglycan component is not possible. It is rather both biopolymers that contribute in a similar fashion to the overall relaxation rate, in particular to the dispersion and the prominence of ^1^H-^14^N cross relaxation with nitrogen nuclei. This has been confirmed by enzymatic treatment with trypsin which reduces the amount of proteoglycans in the sample while leaving the collagen network unaffected. However, a variation of the relaxation times that occurs only after drying and subsequent rehydration is best explained by a differential relaxation behavior of the H_2_O and the residual HDO protons between proteoglycans and collagen, suggesting that in the case of a PG-depleted matrix, water molecules will preferentially interact with the remaining collagen. The interpretation is, however, of a qualitative nature, since the preparation of the in vitro cartilage samples inevitably lead to structural changes during the drying/rehydration cycle which resulted in a 5–10% water loss.

The new information obtained from NMR T_1_ dispersion experiments is essentially derived from the ^2^H properties which are intramolecular in nature; the identical frequency dependence found for ^2^H in D_2_O and for ^1^H in H_2_O molecules confirms that intermolecular contributions to longitudinal relaxation can be neglected in the observed Larmor frequency range of 10 kHz to 30 MHz. This rules out significant contributions of the modulation of dipolar interactions of water protons with protein protons, but also with protons on other water molecules. The dominance of intramolecular interactions is in agreement with the RMTD model (Reorientations Mediated by Translational Displacements) that was originally suggested for inorganic interfaces but has repeatedly been found to also apply in organic systems such as in liquid/gel interactions. RMTD describes the water molecules reorienting due to their free diffusion between PG and collagen strands, but assuming a preferential relative orientation to their surface, for example by directed hydrogen bonds.

This work aims at contributing further evidence about the origin of proton NMR relaxation in cartilage, both as a tool to gain improved understanding of pre-clinical and clinical studies of degenerative diseases such as osteoarthritis, but also as a proxy for other types of biological tissue. Laboratory studies allow the investigation of ^1^H relaxation after partial removal due to D_2_O exchange, or the direct measurement of ^2^H of the latter. In a future study we aim at a more thorough experimental approach towards ^2^H spectroscopy and relaxometry as a function of magnetic field strength, in combination with MRI at high field: analyzing ^2^H relaxation, though not directly available for *in vivo* situations, allows for a much more direct modelling approach to water molecular dynamics due to the single-particle nature of the dominating relaxation processes. RMTD can also be expected to represent a major contribution to relaxation in tissue types more complex than cartilage. Eventually, high-resolution field cycling imaging [56, 57] is the preferred approach to distinguish the different dynamics processes inside the pronounced three-layer structure of mammalian cartilage with its variation of composition and structural ordering between the bone and joint surfaces of cartilage.
